# The matrix glycoprotein Papilin maintains the haematopoietic progenitor pool in *Drosophila* lymph glands

**DOI:** 10.1242/dev.204367

**Published:** 2025-04-10

**Authors:** Jae-In Lee, Sumin Park, Hyunji Park, Youngbin Lee, JinYoung Park, Donghoon Lee, Moon Jong Kim, Kwang-Min Choe

**Affiliations:** ^1^ Department of Systems Biology, Yonsei University, 50 Yonsei-ro, Seodaemun-gu, Seoul 03722, South Korea; ^2^ Department of Life Science, Gachon University, Seongnam 13120, South Korea

**Keywords:** Haematopoiesis, *Drosophila*, Basement membrane, Epidermal growth factor receptor, Wasp

## Abstract

Differentiation of prohaemocytes, the precursors of *Drosophila* blood cells (haemocytes), and the release of haemocytes from the lymph gland, a major larval haematopoietic organ, are vital responses to wasp infestation or tissue degeneration. Although cells and extracellular matrix (ECM) in the lymph gland are known to play a crucial role in haemocyte differentiation, the underlying mechanisms remain unclear. Here, we show that the matrix glycoprotein Papilin (Ppn) is essential for maintaining the prohaemocyte population in lymph glands. In Ppn-depleted larvae, haemocyte differentiation increased with a reduction in the prohaemocyte-containing medullary zone, and lymph gland lobes dispersed prematurely. Ppn was synthesised by plasmatocytes, forming lamellae mainly in the medullary zone. Microbial infection or wasp infestation disrupted the Ppn meshwork within lymph glands. Ppn colocalised with collagen, laminin, nidogen and perlecan. Ppn depletion disrupted the ECM structure, including perlecan organisation. Phenotypes caused by Ppn depletion were partially rescued by perlecan overexpression or inactivation of the epidermal growth factor receptor pathway. Thus, Ppn is crucial for maintaining lymph gland architecture and regulating haemocyte differentiation, highlighting an intricate interaction between the ECM and signalling pathways in haematopoiesis.

## Teaser

**Highlighted Article:** Depletion of Papilin leads to an increase in haemocyte differentiation and a collapse of lymph gland structure, partly through interaction with perlecan and the EGFR signalling pathway, revealing an essential role for Papilin in *Drosophila* blood progenitor maintenance.

## INTRODUCTION

A key aspect of haematopoiesis is balancing the maintenance and differentiation of haematopoietic stem and progenitor cells (HSPCs), akin to other developmental processes. In the bone marrow, HSPCs require proper interactions with neighbouring cells and the extracellular matrix (ECM) for growth, survival and developmental competence (Gao et al., 2018; Mercier and Leonard, 2011; Morrison and Scadden, 2014; Wei and Frenette, 2018). The ECM comprises fibrous proteins, proteoglycans, glycosaminoglycans and glycoproteins, such as collagens, laminins, heparan sulphate and thrombospondins. These are essential components of the stem cell niche and are recognised by various cell surface receptors such as integrins and selectins (Domingues et al., 2017; Klamer and Voermans, 2014; Papy-Garcia and Albanese, 2017). ECM proteins can bind to multiple receptors, and a single receptor can recognise various ECM proteins, facilitating a diverse range of signalling outcomes. Furthermore, each specific interaction may activate distinct signalling cascades, depending on the particular ECM protein and receptor involved. This results in highly flexible and context-specific cellular responses (reviewed by Lee-Thedieck et al., 2022). However, understanding these complex interactions remains challenging due to their combinatorial nature and the diverse composition of niche microenvironments.

*Drosophila* has a robust yet relatively simple immune system, highly conserved with that of mammals (Banerjee et al., 2019; Lemaitre and Hoffmann, 2007). In larvae, haematopoiesis occurs in the lymph gland near the dorsal aorta, consisting of a pair of anterior-most primary lobes and a variable number of smaller posterior lobes (Banerjee et al., 2019; Morin-Poulard et al., 2021) (depicted in Fig. S1A). The primary lobes are organised into three distinct zones: the posterior signalling centre (PSC), the medullary zone (MZ) and the cortical zone (CZ). The PSC, which expresses Knot (also known as Collier) (Crozatier et al., 2004; Oyallon et al., 2016), Antennapedia (Antp) and Hedgehog (Hh) (Mandal et al., 2007), serves as the haematopoietic niche (Mandal et al., 2007; Mondal et al., 2011; Pennetier et al., 2012; Benmimoun et al., 2012; Krzemień et al., 2007; Sinenko et al., 2009), and instructs prohaemocytes to differentiate and disperse during wasp parasitisation (Louradour et al., 2017; Sinenko et al., 2011). The MZ, which expresses *Drosophila* E-cadherin (DE-cad; Shotgun), Thioester-containing protein 4 (TepIV; Tep4) and Domeless (Dome), contains prohaemocytes that are initially active in cell proliferation but become less proliferative as larvae reach the mid-third instar stage (Krzemien et al., 2010a; Jung et al., 2005; Irving et al., 2005). The CZ contains differentiated haemocytes derived from the MZ (Jung et al., 2005; Krzemien et al., 2010a; Minakhina and Steward, 2010). Between the MZ and CZ lies a relatively small group of transitional cells known as intermediate progenitors in a less-well characterised intermediate zone (IZ) (Krzemien et al., 2010b; Sinenko et al., 2009). These cells can be specifically labelled using *CHIZ-GAL4* or *Nplp2-GAL4* (Cho et al., 2020; Spratford et al., 2021). The posterior lobes contain prohaemocytes and function as reservoirs (Kanwal et al., 2021; Jung et al., 2005). Prohaemocytes in the MZ are composed of multiple cell subtypes, distinguished by subtly different gene expression patterns (Girard et al., 2021; Hultmark and Ando, 2022; Cho et al., 2020). Despite this complexity, they remain relatively simple as a group, making them an excellent model for dissecting dynamic interactions during haematopoiesis.

*Drosophila* has three major types of haemocytes. Plasmatocytes, which are small, round and phagocytic, and make up 95% of the total haemocyte population. They uniquely express cell-surface molecules such as Eater and NimC1 (Kocks et al., 2005; Kurucz et al., 2007a,b, 2003; Lebestky et al., 2000; Tepass et al., 1994). Plasmatocytes are responsible for engulfing microbes, apoptotic cells and cellular debris; secreting ECMs to remodel tissues; and attaching to foreign surfaces, such as invading wasp eggs or degenerating self-tissues (Fessler and Fessler, 1989; Lanot et al., 2001; Rizki and Rizki, 1980; Russo et al., 1996). Crystal cells, slightly larger in size, are involved in blood clotting and oxygen transport, comprising 5% of total haemocytes. These cells express prophenoloxidase A1 (ProPOA1; PPO1), Lozenge (Lz) and Hindsight (Hnt; Pebbled) (Shin et al., 2024; Benmimoun et al., 2012; Jung et al., 2005; Lanot et al., 2001; Lebestky et al., 2000; Rizki et al., 1985). Lastly, lamellocytes are large, flat cells that normally appear only during metamorphosis or infection, when they encapsulate foreign or abnormal surfaces of large particles (Rizki and Rizki, 1992; Lanot et al., 2001; Shrestha and Gateff, 1982; Sorrentino et al., 2002). Lamellocytes can be visualised by the expression of the cell-surface antigen Atilla (L1) or by high expression levels of the integrin βPS subunit Myospheroid (Mys) (Honti et al., 2009; Irving et al., 2005).

Shortly after parasitoid wasp infestation, *Drosophila* lymph glands exhibit increased proliferation (Sorrentino et al., 2002; Krzemien et al., 2010a). Lamellocytes are massively induced, and, ultimately, all haemocytes are released into the haemolymph through the dispersal of the lymph gland (Krzemień et al., 2007; Sorrentino et al., 2004; Lanot et al., 2001; Crozatier et al., 2004; Irving et al., 2005). Eggs are initially recognised by haemocytes in an unidentified manner, leading to plasmatocyte migration, adhesion and spreading, and the formation of septate junctions (Irving et al., 2005; Honti et al., 2010; Markus et al., 2009; Mortimer et al., 2013; Russo et al., 1996; Williams et al., 2005). Lamellocytes then bind to the first layer of plasmatocytes, forming multilayered capsules, and the eggs are eventually melanized by prophenoloxidases supplied by the lamellocytes and crystal cells (Vlisidou and Wood, 2015; Shrestha and Gateff, 1982; Rizki and Rizki, 1992; Mortimer et al., 2012; Lanot et al., 2001; Honti et al., 2009). These responses can also target degenerating self-tissues, leading to the formation of melanotic masses, effectively sequestering them (Avet-Rochex et al., 2010; Hanratty and Dearolf, 1993; Kim and Choe, 2014; Minakhina and Steward, 2006; Watson et al., 1991).

The maintenance and differentiation of prohaemocytes in the MZ are regulated by multiple signals from various sources (Morin-Poulard et al., 2021; Banerjee et al., 2019). Maintenance signals include autonomous signals within the MZ from a subset of prohaemocytes expressing Knot (Benmimoun et al., 2015; Oyallon et al., 2016), Wingless signalling and its downstream target DE-cad (Gao et al., 2009, 2013; Jung et al., 2005; Sinenko et al., 2009), and feedback signals from differentiating haemocytes in the CZ (Mondal et al., 2011). Notably, ECM molecules in the lymph glands, such as collagen IV, laminin, Trol (the *Drosophila* perlecan), nidogen (Ndg) and tiggrin, appear to moderate differentiation based on their distribution patterns and the loss-of-function phenotypes of related genes (Dragojlovic-Munther and Martinez-Agosto, 2013; Grigorian et al., 2013; Grigorian et al., 2011b; Khadilkar et al., 2020; Zhang and Cadigan, 2017). Induction signals for prohaemocyte differentiation have also been identified, including PSC-derived reactive oxygen species, which activate both Toll and epidermal growth factor receptor (EGFR) pathways, leading to prohaemocyte differentiation (Holz et al., 2003; Lanot et al., 2001; Louradour et al., 2017; Sinenko et al., 2011; Sorrentino et al., 2002). These signals are triggered upon wasp infestation.

Papilin (Ppn) was first identified as a large, sulphated glycoprotein from the culture media of *Drosophila* Kc cells (Campbell et al., 1987). Ppn contains multiple domains, including TSR and Kunitz, and its structural organisation is well conserved from nematodes to humans (Fessler et al., 2004). Ppn is among the most abundant proteins in the basement membrane, highlighting its role as a core component of this structure. Knockdown of *Ppn* results in defects in muscle development and leads to lethality during embryogenesis and the first instar stage (Campbell et al., 1987; Kramerova et al., 2000; Keeley et al., 2020; Jayadev et al., 2022). In *Caenorhabditis elegans*, Ppn facilitates collagen removal during basement membrane expansion and tissue growth in the gonad (Keeley et al., 2020), and it mediates axon-dendrite adhesion and dendrite patterning (Ramirez-Suarez et al., 2019). *Drosophila* Ppn inhibits procollagen N-proteinase, an ADAMTS metalloproteinase, *in vitro* (Kramerova et al., 2000). However, its role in cell differentiation or immune defence remains unclear.

In this study, we isolated the *Ppn* gene using an RNA interference (RNAi)-based genetic screen and explored its potential roles in *Drosophila* larval lymph glands. We found that Ppn forms a network of interconnected lamellae and septa primarily in the MZ, where it supports ECM structure and suppresses prohaemocyte differentiation by inhibiting EGFR signalling and interacting with perlecan. During bacterial infection or wasp infestation, this Ppn structure collapsed, correlating with prohaemocyte differentiation and lymph gland dispersal. These findings emphasise an essential role of Ppn in maintaining the prohaemocyte population, which is crucial for effective pathogen defence, and support the broader observation that niche ECM is vital for progenitor maintenance.

## RESULTS

### *Ppn* knockdown larvae form melanotic masses

In our previous study, we demonstrated that the basement membrane, particularly laminin, and cell integrity are vital for maintaining self-tolerance and preventing autoimmunity (Kim and Choe, 2014). Building on these insights, we conducted RNAi-based screens to identify genes involved in immunological tolerance. We screened 249 RNAi lines targeting 136 genes encoding ECM proteins, signalling components, and scavenger receptors, using the melanotic encapsulation phenotype as an ‘autoimmune’ assay. Knocking down the integrin βPS (*mys*) gene in haemocytes and fat bodies (*Cg-GAL4, UAS-mys-RNAi*; hereafter *Cg>mys-i*) led to melanotic mass formation in approximately 27% of larvae, establishing a sensitised genetic background for autoimmune responses (Kim and Choe, 2014). To identify potential modifiers, we crossed each *UAS-RNAi* line to the *Cg>mys-i* background, isolating 22 genes that acted as enhancers or suppressors of the melanotic mass phenotype in these larvae (Fig. S1B,C; Table S1). Additionally, a subset of candidate genes was screened by crossing *UAS-RNAi* lines directly with *GAL4* lines, leading to the identification of *Ppn*.

**Fig. 1.**
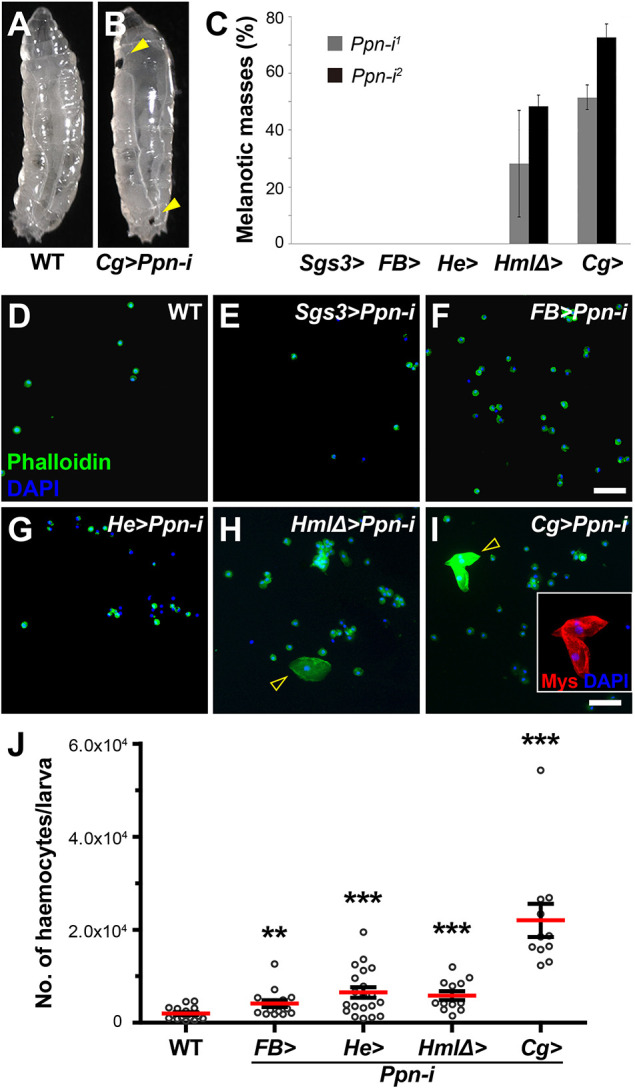
***Ppn* knockdown induces melanotic masse formation.** (A,B) Micrographs of third instar wild-type (WT; *Ore-R*) and *Ppn* RNA interference (RNAi) larvae (*Cg-GAL4 UAS-Ppn-RNAi*, hereafter *Cg>Ppn-i*). The latter shows melanotic masses marked by yellow arrowheads. (C) Percentages of larvae containing melanotic masses in their body cavity, analysed after RNAi knockdown using two independent *UAS-Ppn-RNAi* lines and five different tissue-specific *GAL4* drivers. At least 48 larvae were analyzed for each genotype. (D-I) Analysis of circulating haemocytes in larval bleeds from the indicated genotypes. F-actin was stained with FITC-phalloidin (green), cell nuclei with DAPI (blue) and lamellocytes with anti-Mys antibody (red; inset in I). Lamellocytes are marked with arrowheads in H and I. Scale bars: 50 μm. (J) Quantification of circulating haemocyte numbers in *Ppn*-*RNAi* larvae with different *GAL4* drivers. Statistical significance was determined using the Mann–Whitney *U*-test. **P<0.01, ***P<0.001.

To validate the role of Ppn further, we used three independent *UAS*-*RNAi* lines (*RNAi^1^*, *RNAi^2^* and *RNAi^3^*) targeting different, non-overlapping regions of *Ppn*, and knocked down *Ppn* using tissue-specific or inducible *GAL4* drivers*,* as ubiquitous Ppn depletion is embryonic lethal (Kramerova et al., 2000). Quantitative reverse transcription-polymerase chain reaction (qRT-PCR) confirmed reduction by 60.8% and 39.2% in *Ppn* transcript levels for the first two RNAi lines, respectively, with *Cg-GAL4* drivers (Fig. S1D). Western blot analysis showed the loss of a high molecular weight Ppn band in all three *Ppn* knockdown larvae (Fig. S1E). *Ppn* knockdown larvae (*Cg>Ppn-i*) showed a high percentage of larvae containing melanotic masses in the body cavity (54.3% and 72.6% for *RNAi^1^* and *RNAi^2^*, respectively; Fig. 1A-C). While most melanized masses were found associated with the fat body, a small proportion was observed as haemocyte aggregates.

To determine the primary tissue source of Ppn contributing to the melanotic mass phenotype, we used various tissue-specific *GAL4* drivers. Many ECM molecules are produced by haemocytes and fat bodies (Fessler and Fessler, 1989), consistent with our initial results using the *Cg-GAL4* driver. However, melanotic masses were formed only with *GAL4* lines active in lymph gland haemocytes and mature circulating haemocytes, such as *Hml*Δ*-* and *Cg-GAL4* (Fig. 1C). *GAL4* drivers active in fat bodies (*FB*) or salivary glands (*Sgs3*) did not induce a melanotic mass phenotype with *UAS-Ppn-i* (Fig. 1C). Furthermore, the number of circulating haemocytes significantly increased in these larvae (Fig. 1D-J), and lamellocytes, usually absent until the wandering third-instar stage, were present in the haemolymph of *Ppn* knockdown larvae (Fig. 1H,I, yellow arrowheads), aligning with the melanotic mass phenotype. These findings indicate that haemocyte-derived Ppn is required for the prevention of melanotic mass formation.

### Haemocytes differentiate precociously in the lymph glands of *Ppn* knockdown larvae

Knockdown of *Ppn* did not significantly disrupt tissue integrity in the fat body (see Fig. S2A-E for a comparison with integrin knockdown; Kim and Choe, 2014), suggesting that the melanotic mass phenotype observed in *Ppn* knockdown larvae primarily results from defects in haemocyte function rather than abnormalities in the ‘autoimmune’ target tissues (Watson et al., 1991). To investigate haemocyte abnormalities, we performed a detailed characterisation of the lymph glands of *Ppn* knockdown larvae. We first examined changes in the three types of differentiated haemocytes and the progenitor population.

**Fig. 2.**
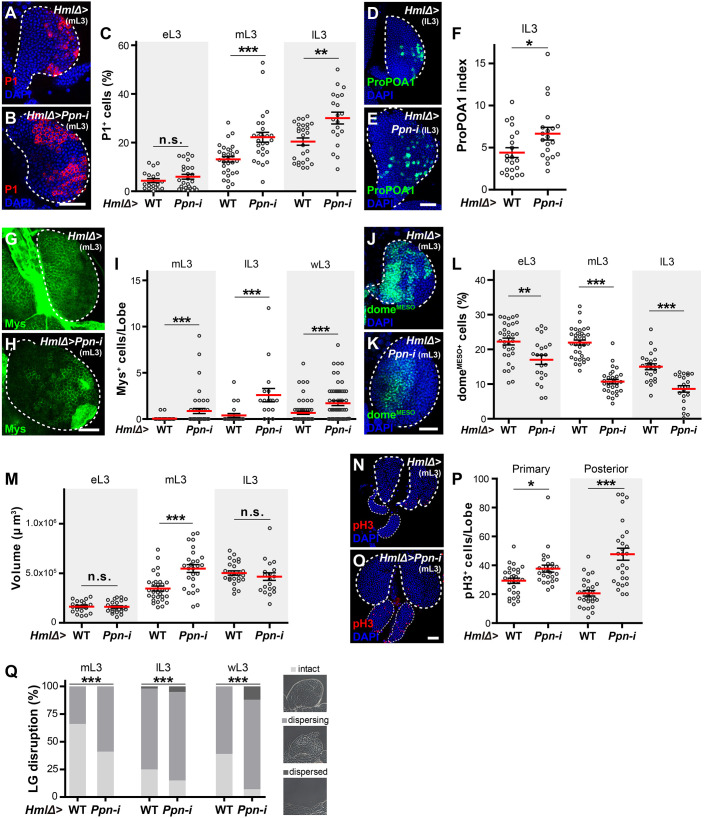
***Ppn* knockdown larvae exhibit precocious haemocyte differentiation and increased proliferation in lymph glands.** (A-P) Lymph glands of control (A,D,G,J,N) and *Hml*Δ*>Ppn-i* (B,E,H,K,O) larvae were analysed using immunohistochemistry. Plasmatocytes were stained with P1 antibody (red; A,B), crystal cells were marked with ProPOA1.sGFP (green; D,E), lamellocytes were stained with anti-Mys antibody (green; G,H), prohaemocytes were marked with dome^MESO^-GFP (green; J,K) and mitotic cells were visualised with anti-phospho-histone H3 (pH3) antibody (red; N,O). Cell nuclei were stained with DAPI (blue). Primary and secondary lobes are delineated by thick and thin dashed lines, respectively. Scale bars: 50 μm. (C,F,I,L) Quantification of the proportion of P1-positive cells (C), ProPOA1.sGFP-positive cell index (F), the number of enlarged Mys-positive cells (I) and dome^MESO^-GFP-positive cell index (L), per primary lobe at the indicated developmental stages are shown. (M) Quantification of the volume of the primary lobe at each developmental stage. (P) Quantification of the number of pH3-positive cells in the primary and posterior lobes. n.s., not significant; *P<0.05, **P<0.01, ***P<0.001 (Mann–Whitney *U*-test). (Q) Histolysis of lymph glands was analysed and quantified into three categories for each primary lobe: intact, dispersing or dispersed (see Materials and Methods; examples shown in images on the right). Quantification of lymph gland dispersal in *GAL4* control and *Hml*Δ*>Ppn-i* larvae is presented as a percentage, with at least 42 lobes measured for each genotype. Pearson's Chi-squared test was used. eL3, mL3, lL3 and wL3- indicate early-, mid-, late- and wandering third instar larvae, respectively. LG, lymph gland.

In control larvae, plasmatocytes, marked with anti-NimC1 antibody (P1) (Asha et al., 2003; Kurucz et al., 2007a), emerged during the early-third instar and gradually increased throughout development until the late-third instar stage, consistent with previous studies (Fig. 2A,C; Fig. S2F,H) (Jung et al., 2005). In *Hml*Δ*>Ppn-i* larvae, the percentages of P1-positive plasmatocytes significantly increased, and the CZ exhibited scattered patterns compared to controls (Fig. 2A-C; Fig. S2F-I). Similar patterns were observed with the other two *UAS-Ppn-i* lines, and with the *Cg-GAL4* driver as well (Fig. S2J-O). The percentage of the area occupied by ProPOA1.sGFP-positive crystal cells nearly doubled in late-third instar *Ppn* knockdown lymph glands (Fig. 2D-F; Fig. S2P-P‴). Moreover, the number of Mys-positive lamellocytes significantly increased in *Ppn* knockdown larvae (Fig. 2G-I; see Materials and Methods). Consistent with this, the percentages of prohaemocytes and width of the MZ, visualised with dome^MESO^-GFP or anti-DE-cad antibodies, respectively, were reduced in the primary lobes of *Hml*Δ*>Ppn-i* larvae (Fig. 2J-L; Fig. S2Q-V).

As regulation of proliferation is also crucial for lymph gland homeostasis, we examined the volume of lymph gland lobes. While no significant differences in primary lobe volume were observed between control and *Hml*Δ*>Ppn-i* larvae at the early-third instar stage, a 1.70-fold increase was evident in *Ppn* knockdown larvae by the mid-third instar stage (Fig. 2M). Concurrently, the number of phospho-histone 3 (pH3)-positive mitotic cells increased in these larvae compared to controls (Fig. 2N-P). At later stages, the volumes of the primary lobes slightly decreased in *Ppn* knockdown larvae, likely due to premature histolysis and dispersal (Fig. 2M,Q). Abnormalities were also observed in secondary lobes upon *Ppn* knockdown; plasmatocytes were present, and both the size and number of mitotic cells were elevated compared to controls (Fig. 2N-P; Fig. S2W-Z). These findings indicate that Ppn normally suppresses prohaemocyte differentiation and over-proliferation in the lymph glands.

### Distribution of Ppn in the lymph glands

**Fig. 3.**
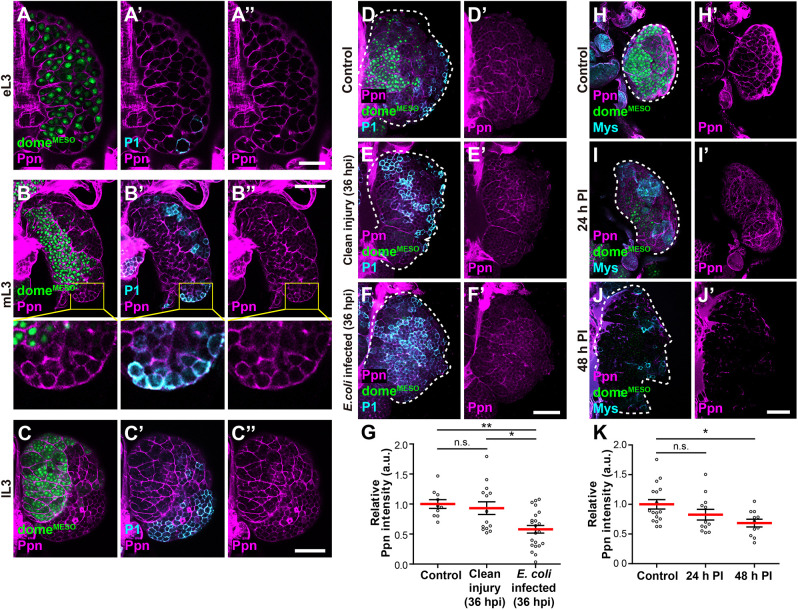
**Ppn forms lamellae prominently in the MZ of lymph glands, with its three-dimensional network disrupted by bacterial infection or wasp infestation.** (A-C″) Distribution of Ppn in the lymph glands of the early- (eL3; A-A″), mid- (mL3; B-B″) and late- (lL3; C-C″) third instar *dome^MESO^-GFP* larvae was visualised using anti-Ppn antisera (magenta). Prohaemocytes were marked with dome^MESO^-GFP (green) and plasmatocytes were stained with P1 antibody (cyan). The regions outlined by yellow squares in (B-B″) are shown at higher magnification below, respectively. (D-F′) Confocal images of the lymph glands of *dome^MESO^-GFP* larvae 120 h after egg laying (AEL). Larvae without injury (D,D′), larvae with clean injury treated with 10% sucrose at 36 h post-injury (hpi; E,E′) and larvae infected with *E*. *coli* at 36 hpi (F,F′) are shown. Ppn, plasmatocytes and prohaemocytes were stained as described for A-C″. (G) Quantification of relative Ppn fluorescence intensity in the lymph glands of the indicated conditions. (H-J′) Confocal images of the lymph glands of *dome^MESO^-GFP* control larvae (H,H′) or 24 h (I,I′) or 48 h (J,J′) post-wasp infestation (PI). Ppn was stained with anti-Ppn antisera (magenta), prohaemocytes were marked with dome^MESO^-GFP (green) and lamellocytes were stained with anti-Mys antibody (cyan). Primary lobes are demarcated with dashed lines. Scale bars: 20 μm (A-A″); 50 μm (B-F′,H-J′). (K) Quantification of relative Ppn fluorescence intensity in the lymph glands of the indicated conditions. *P<0.05, **P<0.01 (Mann–Whitney *U*-test). n.s., not significant; a.u., arbitrary units.

**Fig. 4.**
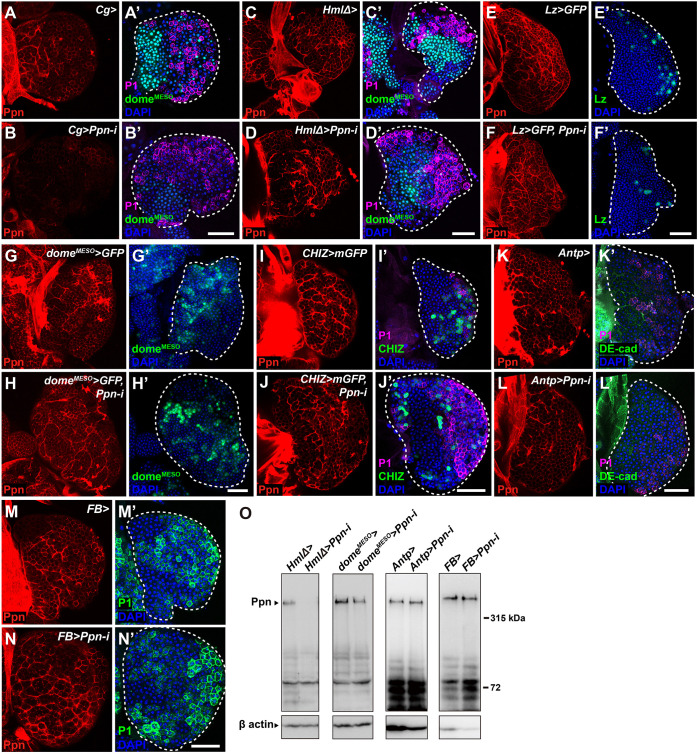
**Ppn is mainly produced by plasmatocytes.** (A-N′) Ppn distribution in late-third instar larval lymph glands was analysed after *Ppn* knockdown using various tissue-specific *GAL4* drivers. Primary lobes are demarcated with dashed lines. *Cg-GAL4* (A-B′), *Hml*Δ*-GAL4* (C-D′), *Lz>GFP* (E-F′), *dome^MESO^>GFP* (G-H′), *CHIZ>mGFP* (I-J′), *Antp-GAL4* (K-L′) or *FB-GAL4* (M-N′) were crossed with *Ore-R* for controls and *UAS-Ppn-i* for tissue-specific knockdown larvae. Ppn was stained with anti-Ppn antisera (red) and plasmatocytes were stained with P1 antibody (magenta) (A′-D′,I′-L′) or green (M′,N′). Prohaemocytes were marked with dome^MESO^ in green (A′-D′,G′,H′), and the MZ was stained with anti-DE-cad antibody in green (K′,L′). Crystal cells (E′,F′) and intermediate progenitors (IPs; I′,J′) were marked with GFP (green). Cell nuclei were stained with DAPI (blue). Scale bars: 50 μm. (O) Ppn protein levels in whole larvae of the indicated genotypes were analysed by western blotting using guinea pig anti-Ppn antisera. β-actin was used as a loading control.

To improve our understanding of the role of Ppn in the lymph gland, we analysed its distribution using immunohistochemistry. To address the limitations of existing rabbit antisera (Campbell et al., 1987), particularly insufficient volume, we generated new, guinea pig anti-Ppn antisera targeting a synthetic peptide from the middle region of the Ppn core protein. Both antisera recognised Ppn protein, but the rabbit anti-Ppn, raised against purified Ppn protein, consistently produced a stronger and more defined staining pattern at the outer basement membrane, likely due to the extensive glycosylation and extracellular localisation of Ppn. The specificity of these antisera was rigorously validated by co-immunostaining and western blotting (see Materials and Methods; Fig. S3A-A″; Fig. S4Q-V′; Fig. 4O). In early-third instar larvae, the primary lobes of the lymph glands were predominantly populated by dome^MESO^-GFP-positive prohaemocytes (Fig. 3A) (Dey et al., 2016; Jung et al., 2005), with a small number of plasmatocytes on the surface (Fig. 3A′). Ppn localised in lamellae, forming numerous septa that accommodated several dome^MESO^-GFP-positive prohaemocytes, resembling the distribution of collagen IV, laminin and Trol (Fig. 3A-A″) (Jung et al., 2005; Grigorian et al., 2011b, 2013; Krzemien et al., 2010b; Khadilkar et al., 2020). As development proceeded, lymph gland lobes enlarged, plasmatocytes increased, and the distinction between the MZ and CZ became clearer (Fig. 3B-C″) (Krzemien et al., 2010b; Jung et al., 2005; Sinenko et al., 2009; Sorrentino et al., 2002). Ppn lamellae in the MZ maintained their thickness and elongated to match the expanding zone. Ppn weakly localised at cell boundaries, with its immunofluorescence increasing in P1-positive plasmatocytes as development progressed (Fig. 3B-C″). Ppn also formed an outer sheath around the primary lobe, visible in the middle plane of confocal sections (Fig. S3B-C′).

We investigated whether immune challenge could alter Ppn distribution within the lymph gland. In *Escherichia coli-*infected larvae, plasmatocytes increased, Ppn meshworks were severely disrupted and lymph gland lobes dispersed, becoming more evident from 24 to 36 h post-injury (hpi; Fig. 3D-G; Fig. S3D-F′) (Khadilkar et al., 2017). The response specificity was analysed by the induction of the antimicrobial peptides *Cecropin A1* (*CecA1*) and *Diptericin A* (*DptA*), measured by qRT-PCR, compared with clean injury controls (Fig. S3G). We further examined whether wasp infestation caused similar effects. Twenty-four hours post-parasitisation, lymph glands showed mildly reduced dome^MESO^-GFP levels compared to controls, indicating prohaemocyte differentiation (Fig. 3H,I; Fig. S3H,I) (Krzemień et al., 2007). Consistent with this, the number of plasmatocytes tended to increase compared to controls, and Mys-positive lamellocytes emerged (Fig. 3H,I; Fig. S3H,I). At this stage, most lymph glands had not ruptured, but the outer sheath of Ppn became irregular and discontinuous (Fig. 3H-I′; Fig. S3J,K). Unlike in uninfested larvae, where thick Ppn lamellae were primarily adjacent to dome^MESO^-GFP-positive progenitor cells, Ppn lamellae were near dome^MESO^-GFP-negative cells or P1-positive plasmatocytes (Fig. S3H-I′, yellow arrowheads), suggesting that Ppn disruption might not be a prerequisite for haemocyte differentiation. Forty-eight hours after parasitism, Ppn outer sheaths were severely disrupted, and its immunofluorescence was significantly reduced, with the residual structure disorganised (Fig. 3H,H′,J-K; Fig. S3H,H′,L,L′). Thus, during microbial infection or wasp infestation, the Ppn meshwork in the lymph glands collapses, correlating with haemocyte differentiation and lymph gland dispersal.

### Ppn is mainly supplied by plasmatocytes

To determine which cell type predominantly produces Ppn, we knocked down *Ppn* in plasmatocytes first using *Cg-GAL4* or *Hml*Δ*-GAL4*, and in crystal cells using *Lz-GAL4*. The lymph glands of *Cg>Ppn-i* larvae were severely disorganised with a near-complete loss of Ppn branches and significantly increased P1-positive plasmatocytes and *Cg-GAL4*-driven EGFP-positive cells at the expense of dome^MESO^-GFP-positive progenitor cells (Fig. 4A-B′; Fig. S4A,B,L-O). The outer sheath of Ppn in the lymph gland lobes was diffuse and often discontinuous (Fig. S4A′,B′). The lymph glands of *Hml*Δ>*Ppn-i* larvae also exhibited decreased Ppn distribution and a significant expansion of *Hml*Δ*-GAL4*-driven EGFP-positive cells in the primary lobes (Fig. 4C-D′; Fig. S4C,D,M,O). However, these defects were less severe than those observed in *Cg>Ppn-i* larvae, possibly due to differences in driver strength. *Ppn* knockdown in crystal cells using *Lz-GAL4* did not significantly affect Ppn distribution or haemocyte differentiation compared to controls (Fig. 4E-F′; Fig. S4M,O). Moreover, the cellular margins around ProPOA1.sGFP-positive cells lacked Ppn staining, reinforcing that crystal cells are not a primary source of Ppn (Fig. S4E,E′).

*Ppn* knockdown using the prohaemocyte-specific *dome^MESO^-GAL4* or *TepIV-GAL4* drivers did not grossly affect haemocyte differentiation or decrease Ppn expression (Fig. 4G-H′; Fig. S4F-G′,L,M,O). However, *Ppn* knockdown using the IZ-specific *CHIZ-GAL4* or *Nplp2-GAL4* drivers caused mild defects in Ppn distribution and haemocyte differentiation, suggesting a partial contribution of differentiating haemocytes to Ppn production (Fig. 4I-J′; Fig. S4H-I′,L,M,O). *Ppn* knockdown using PSC-specific *Antp-GAL4* did not change Ppn distribution and haemocyte differentiation (Fig. 4K-L′; Fig. S4L,O,P). These findings indicate that lymph gland plasmatocytes are the primary Ppn source.

Given the severity differences between *Cg>Ppn-i* and *HmlΔ>Ppn-i* phenotypes, we investigated whether fat bodies also contribute to Ppn production. However, *Ppn* knockdown in fat bodies only (*FB>Ppn-i* or *Lsp2>Ppn-i*) did not decrease Ppn intensity or enhance haemocyte differentiation (Fig. 4M-N′; Fig. S4J-L,O). Interestingly, the distinct intercellular distributions of Ppn in fat bodies, similar to collagen IV (Dai et al., 2017), decreased with *Hml*Δ*-GAL4,* but not with *FB-GAL4* or *Lsp2-GAL4* (Fig. S4Q-W). Western blot analysis of whole larval lysates showed a considerable decrease in Ppn protein levels in *Hml*Δ*>Ppn-i* larvae, similar to *Cg>Ppn-i*, but no changes in *Antp>Ppn-i* or *FB>Ppn-i* larvae (Fig. 4O). Thus, plasmatocytes, not fat body cells, are the major source of Ppn in the lymph glands and fat bodies.

### Ppn is colocalised with collagen, laminin, nidogen and perlecan, and is crucial for their proper organisation within lymph glands

Given that the spatial arrangement of Ppn in lymph glands closely mirrors the patterns of collagen IV meshwork and associated ECM components (Krzemień et al., 2010b; Jung et al., 2005; Grigorian et al., 2013; Khadilkar et al., 2020), we investigated their colocalisation and potential structural interdependence.

**Fig. 5.**
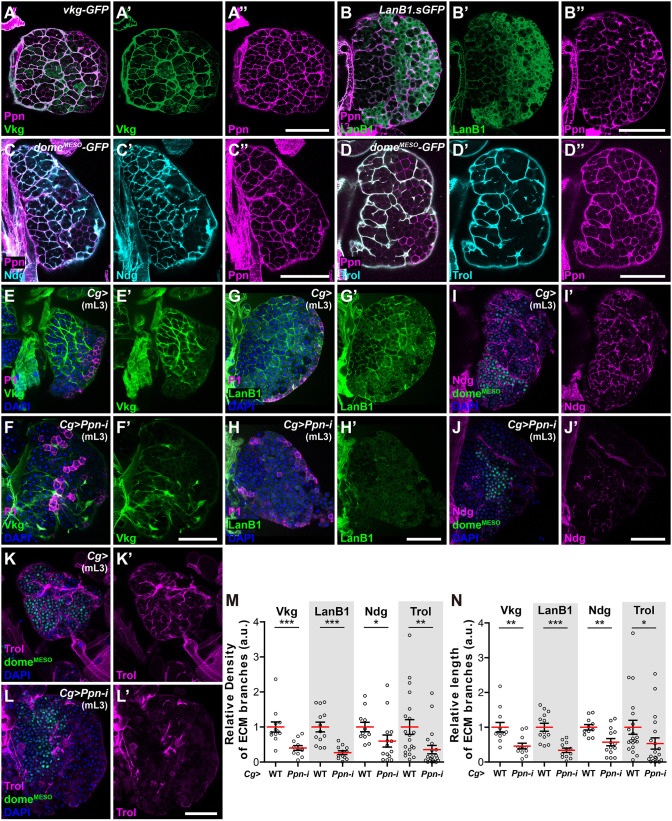
**Organisation of the lymph gland ECM is disrupted in *Ppn* knockdown larvae.** (A-D″) Confocal images of ECM lamellae and Ppn distribution in mid-third instar larval lymph glands. Distribution of collagen (visualised by Vkg-GFP; A,A′, green), laminin (LanB1.sGFP; B,B′, green), nidogen (anti-Ndg antisera; C,C′, cyan) and Trol (anti-Trol antisera; D,D′, cyan) was analysed, compared to that of Ppn (anti-Ppn antisera, magenta). (E-L′) Changes in the distribution of the ECM after *Ppn* knockdown. Collagen (E-F′, green), laminin (G-H′, green), nidogen (I-J′, magenta) and Trol (K-L′, magenta) were examined in controls and *Cg>Ppn-i* larvae. Plasmatocytes were stained with P1 antibody (E-H, magenta) and prohaemocytes were visualised using dome^MESO^-GFP (I-L, green). Scale bars: 50 μm. (M,N) Quantification of the relative density (M) or length (N) of ECM branches around the MZ for the indicated genotypes. *P<0.05, **P<0.01, ***P<0.001 (Mann–Whitney *U*-test). a.u., arbitrary units.

Collagen IV, visualised by Viking-GFP (Vkg-GFP; a protein trap line on *viking*, which encodes the collagen IV α2 chain) (Morin et al., 2001), formed an outer sheath and internal septa within the primary lobes, prominently in the MZ (Fig. 5A,A′), as previously observed (Khadilkar et al., 2020). These patterns largely colocalised with Ppn, although Vkg-GFP did not localise distinctly to individual cell boundaries (Fig. 5A-A″; Fig. S5A-C). We expanded our analysis to include three major components associated with collagen. Laminin, visualised by GFP-tagged LanB1, the β subunit of laminin (LanB1.sGFP) (Sarov et al., 2016), displayed more diffuse patterns in the CZ than Ppn, forming weak, thinner lamellae in the MZ, which moderately colocalised with Ppn (Fig. 5B-B″; Fig. S5D-F). Whereas Vkg-GFP and Ppn formed numerous fine branches in the outer plane of the lobe, LanB1.sGFP branches were mainly localised to the middle plane of the lobe based on *z*-sectioning by confocal microscopy (Fig. 5A-B″; Fig. S3B). Ndg and Trol, visualised by immunohistochemistry with anti-Ndg and anti-Trol antisera, respectively, exhibited a highly branched meshwork in the dome^MESO^-positive MZ and also encased the lobe (Fig. 5C-D″), as reported earlier (Grigorian et al., 2013). These patterns highly overlapped with those of Ppn, except that Ppn in the individual cell boundaries extended to the periphery of CZ, while Ndg or Trol immunostaining was often excluded from large clusters of plasmatocytes (Fig. 5C-D″; Fig. S5G-L).

To investigate structural dependence, we analysed the distribution of collagen, laminin, nidogen and perlecan in the larval lymph glands of *Cg>Ppn-i* at mid- (Fig. 5E-L′) and late- (Fig. S5M-T′) third instar stages. In *Ppn* knockdown larvae, the lamellae structures highlighted by Vkg-GFP and LanB1.sGFP were noticeably disorganised, and the density and length of ECM branches were reduced (Fig. 5E-H′,M,N; Fig. S5M-P′). In *Cg>Ppn-i* larvae, ECM collapse and reduced branch density and length were also revealed by Ndg and Trol immunostaining (Fig. 5I-N; Fig. S5Q-T′). To determine whether the reduction of ECM branches in *Ppn* knockdown lymph glands resulted directly from Ppn breakdown or indirectly from MZ reduction, we examined the Trol and Vkg lamellae in the remaining MZ of *Ppn* knockdown lymph glands. Breakdown of Trol branches was observed in the situation where Vkg-GFP branches and DE-cad-labelled MZ remained substantially, suggesting that Trol breakdown was due to Ppn depletion and not caused by a secondary effect of MZ disappearance (Fig. S5U-V′). These results indicate that Ppn is necessary for the proper organisation of all four major ECM components in the lymph glands.

### Ppn functions upstream of Trol in lymph gland organisation

**Fig. 6.**
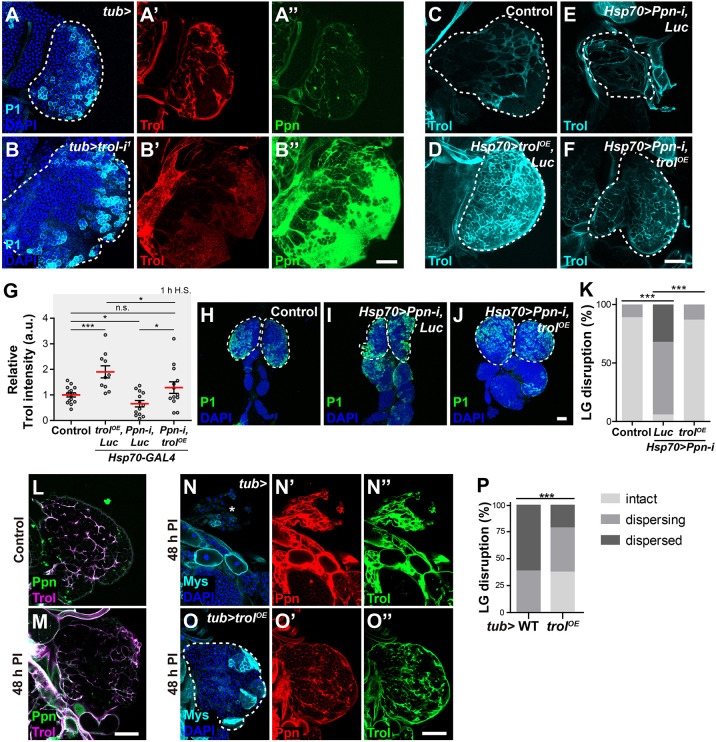
**Ppn does not structurally depend on Trol, and Trol overexpression partially suppresses the haematopoietic defects in *Ppn* knockdown lymph glands.** (A-B″) Distribution of Trol (anti-Trol antisera; red) and Ppn (anti-Ppn antisera; green) in *tubGAL80^ts^/+; tubP-GAL4*, used as controls (hereafter *tub-GAL4*; A-A″) and *tub>trol-i^1^* (B-B″). Plasmatocytes were stained with P1 antibody (cyan), and cell nuclei were stained with DAPI (blue). (C-K) Rescue effects of *trol* overexpression on *Ppn* knockdown lymph glands, focusing on Trol distribution (C-G) and lymph gland dispersal (H-K). *UAS-Ppn-i*-only was used as a control. Crosses were performed with *Hsp70-GAL4*, followed by heat shock (H.S.) for 1 h and dissection after 24 h (see Materials and Methods). These included *UAS-trol^OE^, Luciferase* (*Luc*); *UAS-Ppn-i, Luc*; and *UAS-Ppn-i, trol^OE^*. *Luc* was used to normalise *UAS* dosage. Trol distribution was visualised with anti-Trol antisera (C-F, cyan), with quantification of relative Trol fluorescence intensity (G). n.s., not significant. *P<0.05, ***P<0.001 (Mann–Whitney *U*-test). (H-K) Lymph gland dispersal was quantified as percentage of each category (see Materials and Methods) (K), with at least 27 lobes measured for each genotype. Plasmatocytes were stained with P1 antibody (green), and cell nuclei were stained with DAPI (blue; H-J). a.u., arbitrary units; LG, lymph gland. (L,M) Confocal images of *Hml.DsRed* lymph glands at 123 h AEL (L) and 48 h post-wasp infestation (PI) (M). Ppn and Trol were stained with anti-Ppn (green) and anti-Trol (magenta) antisera, respectively. (N-O″) Confocal images of lymph glands from *tub-GAL4* controls (N-N″) and *tub>trol^OE^* (O-O″) 48 h PI. Ppn and Trol were stained with anti-Ppn (red) and anti-Trol (green) antisera, respectively. Lamellocytes were stained with anti-Mys antibody (cyan) and cell nuclei were stained with DAPI (blue). Primary lobes are demarcated with white dashed lines and asterisk indicates completely dispersed primary lobes. (P) Quantification of the dispersal of late-third instar larval lymph gland, presented as percentage of each category with at least 34 lobes measured per genotype. ***P<0.001 (Pearson's Chi-squared test). Scale bars: 50 μm.

Since Trol depletion leads to lymph gland defects similar to those observed with *Ppn* (Grigorian et al., 2013; Dragojlovic-Munther and Martinez-Agosto, 2013), we further examined the relationship between Ppn and Trol. Ubiquitous knockdown of *trol* led to a marked increase in Ppn protein levels, especially in the CZ, likely due to a rise in the number of plasmatocytes capable of producing Ppn (Fig. 6A-B″; Fig. S6B). Ppn lamellae were still observed in the MZ even when Trol protein levels were severely reduced and its distribution patterns were disrupted. Similar results were obtained with another *trol-RNAi* (Fig. S6A-B). These findings indicate that Ppn distribution is not dependent on Trol.

We also examined the effects of *trol* overexpression (OE) on the *Ppn* knockdown phenotypes. First, *trol* overexpression in a wild-type background significantly increased Trol immunofluorescence levels and lamellae in lymph glands, as expected (Fig. 6C,D,G). Second, *trol* OE in *Ppn* knockdown larvae restored the three-dimensional meshwork of Trol. However, compared to controls, Trol immunofluorescence in the lymph glands of *Hsp70>Ppn-i, trol^OE^* larvae did not significantly increase (Fig. 6C-G). These results are consistent with the notion that proper organisation of Trol requires Ppn. Third, *trol* OE in *Ppn* knockdown larvae restored the integrity of lymph gland lobes and significantly reduced their dispersal (Fig. 6H-K; Fig. S6C-F). However, there was no noticeable difference in plasmatocyte differentiation compared to *Ppn* knockdown alone. Collectively, we conclude that Ppn functions upstream of Trol in the structural organisation of lymph glands.

Since Trol acts downstream of Ppn in lymph gland organisation, we tested this hypothesis in the context of wasp infestation to determine whether *trol* OE could inhibit lymph gland dispersal. Wasp infestation led to the breakdown of Trol lamellae (Fig. 6L,M), as previously reported (Louradour et al., 2017). *trol* OE suppressed lymph gland dispersal, supporting the structural relationship between Ppn and Trol (Fig. 6N-P). Furthermore, our findings corroborate that disruption of the lymph gland ECMs is necessary for defending against wasp infestation.

### Ppn regulates the differentiation of prohaemocytes partly via the EGFR signalling pathway

To explore the mechanisms by which Ppn modulates haemocyte differentiation, we examined several signalling pathways known to affect lymph gland homeostasis and tested whether manipulating them could suppress *Ppn*-knockdown phenotypes (Louradour et al., 2017; Mondal et al., 2011; Sinenko et al., 2009, 2011; Yu et al., 2018; Zettervall et al., 2004).

**Fig. 7.**
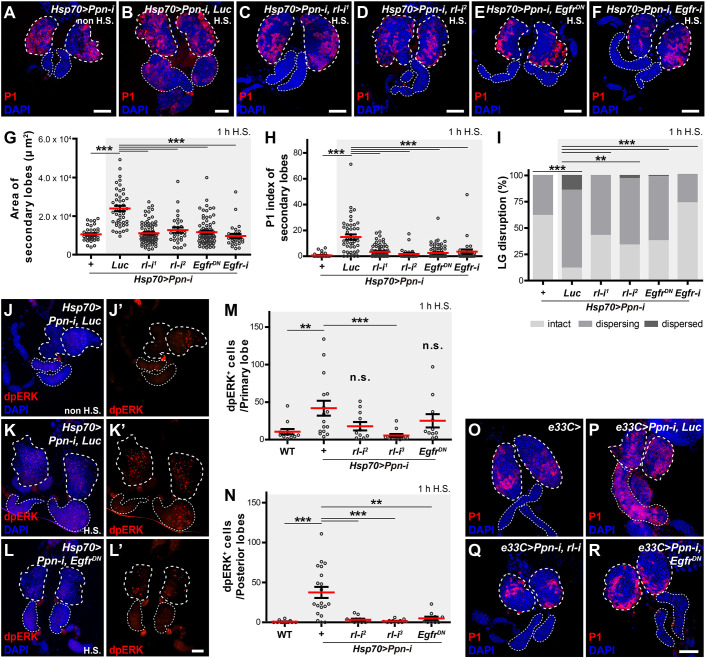
**Ppn regulates lymph gland haematopoiesis by suppressing EGFR pathway activation.** (A-F) EGFR pathway suppression in *Ppn* knockdown larvae was achieved using multiple strategies. *Hsp70>Ppn-i* without heat shock (H.S.) was used as a control (A). For B-F, the following crosses were performed with *Hsp70-GAL4* and treated with 1 h H.S.: *UAS-Ppn-i, Luc*; *UAS-Ppn-i*, *rl-i^1^*; *UAS-Ppn-i*, *rl-i^2^*; *Ppn-i, Egfr^DN^*; and *UAS-Ppn-i*, *Egfr-i*. Plasmatocytes were stained with P1 antibody (red) and cell nuclei with DAPI (blue) at the wandering third instar stage. (G,H) Quantification of lobe size (G) and P1-positive cell index (H) per secondary lobe. *Hsp70>Ppn-i* without H.S., were used as a control. Genotypes within the grey shaded area represent samples treated with 1 h H.S. to induce GAL4 expression. ***P<0.001 (Mann–Whitney *U*-test). (I) Quantification of the dispersal of late-third instar larval lymph gland, presented as percentage of each category (see Materials and Methods). At least 64 lobes were measured per genotype. **P<0.01, ***P<0.001 (Pearson's Chi-squared test). (J-L′) EGFR pathway activity was evaluated by anti-dpERK antibody staining (red) in the lymph glands of the wandering third instar stage. *Hsp70>Ppn-i, Luc* without H.S. (J), *Hsp70>Ppn-i, Luc* with H.S. (K) and *Hsp70>Ppn-i, Egfr^DN^* with H.S. (L) were analysed. Cell nuclei were stained with DAPI (blue). (M,N) Reduced dpERK-positive cell number in primary (M) and posterior (N) lobes after EGFR blockade in *Ppn* knockdown larvae. n.s., not significant; **P<0.01, ***P<0.001 (Mann–Whitney *U*-test). (O-R) The suppressive effects of EGFR pathway blockade on *Ppn* knockdown larvae were re-confirmed using the *e33C-GAL4* driver in *e33C-GAL4/+* (O), *e33C>Ppn-i, Luc* (P), *e33C>Ppn-i, rl-i* (Q) and *e33C>Ppn-i, Egfr^DN^* (R). Plasmatocytes and cell nuclei were stained as described for A-F. Primary and secondary lobes are demarcated with thick and thin dashed lines, respectively. Scale bars: 50 μm.

We found that inhibiting EGFR signalling by RNAi-mediated knockdown of *rl* (*rolled*; encoding *Drosophila* MAPK/ERK) or *Egfr*, or by overexpressing a dominant-negative form (DN) of *Egfr,* rescued several phenotypic defects in *Ppn* knockdown larvae (*Ppn* knockdown was induced using heat-inducible, ubiquitous *Hsp70-GAL4*) (Fig. 7A-F). Although the primary lobes still showed abnormalities, the size and haemocyte differentiation in the secondary lobes were restored to normal levels (Fig. 7G,H). Furthermore, blocking the EGFR pathway prevented premature histolysis of the primary lobes in *Ppn* knockdown larvae (Fig. 7I). These findings suggest that EGFR signalling is abnormally activated in *Ppn* knockdown lymph glands. Supporting this, *Ppn* knockdown lymph glands exhibited elevated levels of dual-phosphorylated ERK (dpERK) in both primary and posterior lobes (Fig. 7J-K′,M,N; Fig. S7A-B′). Notably, inhibiting EGFR signalling reduced dpERK levels in the primary lobes and significantly suppressed them in the posterior lobes (Fig. 7K-N). These results indicate that *Ppn* knockdown leads to derepression of the EGFR signalling pathway.

To further test whether the *Ppn* knockdown phenotypes could be suppressed by tissue-specific inhibition of EGFR signalling, we first showed that ectopic expression of an activated form (act) of EGFR induced haemocyte differentiation phenotypes when driven in the MZ, but not in the CZ, of lymph glands (Fig. S7C-H), consistent with recent findings (Cho et al., 2024). This suggests that the prohaemocyte population, rather than differentiated haemocytes, is competent to EGFR signalling, leading to premature differentiation. Thus, suppressing EGFR signalling only in the CZ failed to rescue the *Ppn* knockdown phenotypes (Fig. S7I-O). Using *e33C-GAL4*, a pan lymph-gland *GAL4* driver, we were able to show that blocking EGFR signalling suppressed *Ppn* knockdown phenotypes (Fig. 7O-R) (Harrison et al., 1995). Finally, employing a combined MZ-CZ *GAL4* driver yielded essentially the same results (Fig. S7P-R). Overall, we conclude that Ppn regulates prohaemocyte maintenance partly by inhibiting EGFR signalling activation in the MZ.

## DISCUSSION

The composition of the haematopoietic niche is crucial for maintaining an adequate supply of progenitor cells and preventing their depletion. In this study, we found that the *Drosophila* ECM glycoprotein Ppn forms lamellae and septa that associate more intimately with progenitor cells in the MZ and less prominently in the CZ, which is reminiscent of previous reports on basement membrane distribution in this organ (Grigorian et al., 2011a, 2013). Ppn colocalises with collagen, laminin, nidogen and perlecan, and also forms an outer sheath encasing the lobe. The three-dimensional Ppn meshwork is disrupted at the onset of metamorphosis or wasp infestation, similar to what has been observed in lymph gland ECMs (Grigorian et al., 2011a; Lanot et al., 2001; Sorrentino et al., 2002). The phenotypes displayed by Ppn-deficient larval lymph glands share many aspects with those lacking Vkg, Trol, Ndg or the accessory protein tiggrin, particularly the precocious differentiation of plasmatocytes (Fogerty et al., 1994; Grigorian et al., 2013; Khadilkar et al., 2020), supporting the idea that lymph gland ECMs maintain the prohaemocyte pool.

How does Ppn regulate prohaemocyte differentiation? Ppn and the other ECM components may collectively inhibit this process via cellular receptors such as integrins on prohaemocytes. Integrin βPS is highly expressed in the MZ, and its depletion in the MZ, but not the CZ, selectively leads to prohaemocyte differentiation in the lymph glands (Khadilkar et al., 2020). Upon immune challenge or developmental signals, Ppn may be disrupted in an instructive manner, triggering the differentiation pathway. Alternatively, Ppn might function as a meshwork of latches that passively block haemocyte differentiation. In this scenario, the balance between inducers and latches would determine the state of cell differentiation. The frequent presence of Ppn lamellae near local differentiation sites during wasp infestation supports this hypothesis. In this case, differentiated haemocytes may secrete proteinase to degrade matrix proteins, including Ppn.

Our results indicate that larval Ppn is primarily synthesised by a subset of plasmatocytes, and possibly by some cells in the IZ. Since Ppn lamellae are mainly observed in the MZ, it suggests that Ppn synthesised by plasmatocytes is trapped in the MZ, forming lamellae and septa, and even accumulating on fat bodies to create collagen IV intercellular concentration-like deposits (Dai et al., 2017). This contrasts with the general finding that both haemocytes and fat bodies contribute to *Drosophila* larval basement membranes (Fessler and Fessler, 1989; Pastor-Pareja, 2020). However, it aligns with findings that the *de novo* formation of the embryonic basement membrane is largely contributed to by embryonic haemocytes rather than fat bodies (Matsubayashi et al., 2017). Similarly, migrating embryonic haemocytes produce laminins as part of their migratory trails, reinforcing directional and effective migration (Sánchez-Sánchez et al., 2017). Ppn forms prominent lamellae in the MZ, possibly due to the selective presence of its cellular receptors in that area. Lymph glands have small cell populations that are currently not well characterised (Baldeosingh et al., 2018; Benmimoun et al., 2015; Dragojlovic-Munther and Martinez-Agosto, 2013; Krzemień et al., 2010b; Sinenko et al., 2009), and identifying the subgroup that supplies Ppn and examining its regulation during haematopoiesis will be interesting.

We found that Ppn is required for the proper organisation of Trol but not vice versa. Ppn is also required for the normal distribution of the other three major components of the basement membrane to varying degrees. However, the possibility of interdependence among these components has not been tested. During *de novo* synthesis of the basement membrane, a structural hierarchy exists among its major components, with laminin typically acting as the initiator and collagen IV as the most abundant protein (Pastor-Pareja, 2020; Matsubayashi et al., 2017). Perlecan, nidogen and other associated components confer or modulate the properties of the meshwork. For example, *Drosophila* Trol and *C. elegans* Ppn facilitate collagen IV removal during tissue growth, allowing for basement membrane expansion (Pastor-Pareja and Xu, 2011; Keeley et al., 2020). In *Drosophila*, nidogen serves as a link between laminin and collagen IV networks (Dai et al., 2018; Wolfstetter et al., 2019). Trol is also known to inhibit fibroblast growth factor ligands and promote the Hh signalling pathway, thereby maintaining the prohaemocyte population (Dragojlovic-Munther and Martinez-Agosto, 2013; Grigorian et al., 2013). In this context, Ppn may be crucial as a key regulator in both development and pathogen defence, particularly in dynamic changes to basement membrane properties and cell signalling.

The sharp increase in Ppn levels in *trol* knockdown lymph glands is intriguing. This change could result from plasmatocyte expansion, or it may arise from an interplay between Ppn, Trol and other ECM components. Given that both Trol and Ppn interact closely with collagen, Ppn may have increased binding opportunities following Trol depletion. Alternatively, this interaction could be mediated through a shared receptor (Pastor-Pareja and Xu, 2011; Keeley et al., 2020; Hayes et al., 2022). Further studies are required to clarify this mechanism.

We also discovered that depleting Ppn derepresses the activation of the EGFR signalling pathway in the lymph glands, revealing a molecular mechanism underlying the Ppn loss-of-function phenotype. Wasp infestation triggers reactive oxygen species production and the secretion of one of the four known EGFR ligands, Spitz (Spi), in the PSC, which in turn activates the EGFR pathway in the MZ (Louradour et al., 2017; Sinenko et al., 2011). Ppn may play a role in sequestering EGFR ligand(s), creating a reservoir in the MZ. Our attempts to rescue the *Ppn* knockdown phenotype by removing *spi* were unsuccessful (data not shown), suggesting that the underlying mechanism may be more complex, involving multiple ligands or acting downstream of the receptor level.

Kramerova et al., who originally purified and characterised Ppn molecules in *Drosophila*, reported that Ppn and other ECM proteins are released from freshly collected haemocytes that strongly adhere to foreign surfaces (see discussion in Kramerova et al., 2000). In this study, we focused on the role of Ppn in haematopoietic organs. Given the formation of melanotic masses in *Ppn* knockdown larvae, it is plausible that Ppn may also play a role in self/non-self discrimination, potentially serving as a self-tolerance tag. Future research should explore its potential functions in peripheral tissues.

## MATERIALS AND METHODS

### Fly strains

For this study, the following lines were obtained from public stock centres: *UAS-Ppn-i* [stock numbers 108005 (*Ppn-i^1^*) and 16523 (*Ppn-i^2^*)], *UAS-Egfr-i* (43267) and *ProPOA1.sGFP* (318298) from the Vienna *Drosophila* RNAi Center (VDRC); *Oregon R*, *He-GAL4* (8699), *Hml*Δ*-GAL4* (30139 and 30141), *Sgs3-GAL4* (6870), *Cg-GAL4* (7011), *Hsp70-GAL4* (1799), *Lsp2-GAL4* (6357), *tubGAL80ts;tubP-GAL4* (86328), *UAS-mys-i* (29619), *UAS-EGFP* (5430), *UAS-Luciferase* (35788), *UAS-trol-i* [38298 (*trol-i^1^*) and 29440 (*trol-i^2^*)], *UAS-trol^OE^* (65274), *UAS-rl-i* [34855 (*rl-i^1^*), 31524 (*rl-i^2^*) and 31387 (*rl-i^3^*)], *UAS-Egfr^DN^* (5364), *UAS-Egfr^λ^* (59843; *Egfr^act1^*), *UAS-Egfr^CA^* (9533; *Egfr^act2^*) and *vkg-GFP* (98343) from the Bloomington *Drosophila* Stock Center; *UAS-Ppn-i* (18436R-4; *Ppn-i^3^*) from the National Institute of Genetics (NIG); and *TepIV-GAL4* (105442) from the *Drosophila* Genomic Research Center (Japan). The following stocks were kindly provided from private collections: *FB-GAL4* by R. P. Kuhnlein (Gronke et al., 2003); *LanB1.sGFP* (VDRC, 318180) by J. Pastor-Pareja (Instituto de Neurociencias, Spain); *Elav-GAL80;dome^MESO^>GFP/TM6B*, *dome^MESO^-GFP*, *UAS-EGFP/FM7i;Nplp2-GAL4/CyO* (Korea *Drosophila* Resource Center, 10023), *Hml-DsRed/CyO,Tb^1^*, *Lz>GFP* and *Antp-GAL4* by J. Shim (Seoul National University, Korea); *e33C-GAL4/TM6c* by D. Ferrandon (Université de Strasbourg, France); *CHIZ-GAL4,UAS-mGFP* by U. Banerjee (University of California, USA). The stock numbers of the modifier screen are listed in Table S1.

### Conditions for rearing and heat-shock treatment of *Drosophila*

Flies were raised at 25°C on standard cornmeal and agar medium. For experiments involving melanotic mass formation, flies were reared on a conditioned medium consisting of 5% (w/v) dry yeast, 3.53% (w/v) starch, 0.51% (w/v) agar and 7% (w/v) dextrose, as melanotic mass formation is influenced by the culture medium (Asano and Takebuchi, 2009; Burnet and Sang, 1964). Larvae were staged according to developmental time points at 25°C as follows: early-third larval instar (eL3): 78 h after egg laying (AEL); mid-third larval instar (mL3): 99 h AEL; late-third larval instar (lL3): 120 h AEL. Since *Cg>Ppn-i* larvae exhibited severe developmental delays, their stages were determined based on morphological features, with 3, 12, 20 and 24 additional hours added to the normal time periods for eL3, mL3, lL3 and wandering third (wL3) larval instars, respectively. For experiments using *Hsp70-GAL4*, eggs were collected for 6 h, and vials were kept at 25°C. At 96 h AEL, vials were submerged in a 37°C water bath for 1 h, then returned to 25°C until inspection or dissection. For all experiments with the *tubGAL80ts/+; tubP-GAL4* (*tub-GAL4*) line, flies were raised at 25°C without any temperature shifts.

To knock down *trol* within the lymph glands, only ubiquitously expressed GAL4 drivers (*Hsp70-GAL4* and *tubGAL80ts/+; tubP-GAL4*) effectively disrupted Trol expression. Therefore, we used these ubiquitous drivers rather than zone-specific GAL4 drivers for *trol* knockdown.

### Quantitative PCR

Total RNA was extracted from ten whole larvae at the wandering third instar stage using TRIzol reagent (15596026, Invitrogen), treated with DNase I (89836, Thermo Fisher Scientific), and quantified. cDNA was synthesised from 2 μg RNA using Moloney murine leukemia virus reverse transcriptase (M-MLV RT; M1701, Promega). Quantitative real-time PCR was performed in quadruplicate using TB Green Premix Ex Taq (Tli RNaseH Plus; RR420A, Takara Bio) with CFX duet (Bio-Rad), and *β-tubulin* was used to normalise RNA levels. Relative mRNA levels were calculated using the comparative cycle threshold (Ct) method. The following primers were used: 5′-CTGCCAGTGAAAATCTGCC-3′ and 5′-TCTTGTTGCCCTTGCATCC-3′ for *Ppn*; 5′-ATCATCACACACGGACAGGA-3′ and 5′- GAGCTGGATGATGGGGAGTA-3′ for *β-tubulin*; 5′-ATGCAGTTCACCATTGCCGTC-3′ and 5′-TCCAGCTCGGTTCTGAGTTG-3′ for *DptA*; and 5′-ATGAACTTCTACAACATCTT-3′ and 5′-GGCAGTTGCGGCGACATTGG-3′ for *CecA1*.

### Western blotting analysis

For western blotting, ten wandering third instar larvae per genotype were homogenised in lysis buffer [50 mM Tris-HCl (pH 7.4), 150 mM NaCl, 1 mM EDTA, 0.5% (w/v) NP-40 and 1/250 protein protease inhibitor cocktail] and cleared by centrifugation at 13,000 ***g*** for 10 min at 4°C. Lysates were mixed with 2× Laemmli sample buffer, boiled for 7 min at 99°C, separated on a polyacrylamide gel (4-15%; 4561086, Bio-Rad), and transferred onto nitrocellulose membranes using a wet transfer system. Membranes were blocked with 5% (w/v) skimmed milk in TBST [10 mM Tris (pH 8.0), 150 mM NaCl and 0.05% (v/v) Tween 20] for 1 h, then probed with primary antibodies overnight at 4°C. After washing thrice with TBST, membranes were incubated with horseradish peroxidase-conjugated secondary antibodies in TBST with 1% (w/v) skimmed milk for 1h. Following four washes with TBST, membranes were visualised using the WEST-ZOL Plus Western blot detection system (16024, iNtRon) and ImageQuant LAS 4000 (GE Healthcare). Primary antibodies used were: rabbit anti-Ppn (1:5000; a gift from C. von Bredow, Technical University of Dresden, Germany), guinea pig anti-Ppn (1:20,000; custom-produced; see ‘Antibody production’ section for details) and rabbit anti-β-actin (1:10,000; 4967, Cell Signaling Technology). Secondary antibodies used were: horseradish peroxidase-conjugated anti-rabbit (1:5000; sc-2004, Santa Cruz Biotechnology) and anti-guinea pig (1:5000; sc-2438, Santa Cruz Biotechnology) antibodies.

### Melanotic mass counting

Eggs were collected and larvae were raised in the conditioned media described above. To control for crowding, no more than 50-100 eggs were placed in each vial, as melanotic mass formation is influenced by density (Kim and Choe, 2014). Melanotic masses were counted during the wandering and white pupal stages. The percentage of larvae or pupae with at least one melanotic mass was calculated. Three or more vials were used for each genotype.

### Genetic modifier screening

We generated a second chromosome recombinant strain of *Cg-GAL4* and *UAS-mys-i*. Five to six virgin females of *UAS-RNAi* lines or *UAS* overexpression lines were crossed with two to three males of *Cg>mys-i*, and the vials were flipped every 3 days. Wandering third instar larvae were collected and examined for melanotic masses. To minimise false positives from off-target RNAi effects, multiple RNAi strains were tested when possible. Cut-off values were arbitrarily set at 70% for enhancer candidates and 10% for suppressor candidates. Genes were classified as enhancers or suppressors if all tested RNAi lines exhibited consistent trends.

### Circulating haemocyte counting

Wandering third instar larvae were collected and rinsed with distilled water. A single larva was bled onto a silicone pad into a 12 µl drop of PBS. A droplet of diluted haemolymph was transferred onto a Neubauer haemocytometer using a micropipette tip. Haemocytes were counted based on morphology. At least ten larvae were analysed per genotype.

### Immunohistochemistry, antibodies and imaging

To image circulating haemocytes, wandering third instar larvae were washed, dissected on a glass slide in PBS, and allowed to adhere for 30 min before fixation with 4% (w/v) paraformaldehyde (PFA) for 30 min at room temperature. For lymph glands, third instar larvae were dissected on a silicone pad in PBS, and the internal organs were transferred to 4% PFA and fixed for 30 min at room temperature. Samples were washed thrice with 0.3% (v/v) Triton X-100 in PBS (PBST) and incubated in a blocking solution of 5% (v/v) normal goat serum diluted in PBST (NGS PBST) for 45 min. Samples were then incubated with primary antibodies in NGS PBST for 2 h at room temperature and washed thrice with PBST. Secondary antibodies were applied in NGS PBST for 2 h at room temperature, followed by washing and mounting on VECTASHIELD with 4′,6-diamidino-2-phenylindole (DAPI) (Vector Laboratories, Inc.). Imaging was performed using fluorescence (Zeiss Axio Imager 2) or confocal (Zeiss LSM880 or Zeiss LSM900) microscopy. For fat bodies, 0.2% (v/v) Tween 20 was used instead of 0.3% Triton X-100 in PBST and NGS PBST.

The following primary antibodies were used: mouse anti-P1 (1:100; a gift from I. Ando) (Asha et al., 2003; Kurucz et al., 2007a), mouse anti-Hnt (1:100; Developmental Studies Hybridoma Bank, DSHB), rat anti-DE-cadherin (1:50; DSHB), mouse anti-Mys (1:200; DSHB), rabbit anti-Ppn (Campbell et al., 1987) (1:500; a gift from C. von Bredow; the antisera have been used successfully to stain haemocytes of the black soldier fly) (von Bredow et al., 2022), guinea pig anti-Ppn (1:500; referred to as Ppn GP; unless otherwise specified, this antiserum was used for Ppn immunostaining), rabbit anti-Ndg (1:150; a gift from A. Holz) (Wolfstetter et al., 2009), rabbit anti-Trol (1:500; a gift from A. Gonzalez-Reyes) (Diaz-Torres et al., 2021), phalloidin-FITC (1:100; P5282, Sigma-Aldrich), rabbit anti-phospho-histone 3 (1:100; H0412, Sigma-Aldrich) and rabbit anti-phospho-p42/44 MAPK (1:100; 4370, Cell Signaling Technology). The following secondary antibodies were used: Alexa 546-conjugated goat anti-mouse IgG (1:500; A-11003, Invitrogen), Alexa 488-conjugated goat anti-mouse IgG (1:500; A-11001, Invitrogen), Alexa 647-conjugated goat anti-mouse IgG (1:500; A-21235, Life Technologies), Alexa 488-conjugated goat anti-rabbit IgG (1:500; A-11008, Molecular Probes), Alexa 546-conjugated goat anti-rabbit IgG (1:500; A-11010, Molecular Probes), Alexa 633-conjugated goat anti-rabbit IgG (1:500; A-21070, Invitrogen), Alexa 488-conjugated goat anti-rat IgG (1:500; A-11006, Invitrogen), Alexa 546-conjugated goat anti-rat IgG (1:500; A-11081, Invitrogen), Alexa 488-conjugated goat anti-guinea pig IgG (1:500; A-11073, Life Technologies) and Alexa 546-conjugated goat anti-guinea pig IgG (1:500; A-11074, Invitrogen) antibodies.

For immunostaining of DE-cadherin and guinea pig anti-Ppn, lymph glands were incubated with anti-DE-cadherin antibody (1:50) or anti-Ppn antibody (1:500) in 1× PBS (pH 7.2) before fixation (Langevin et al., 2005) for 1 h at 4°C. Tissues were then fixed in 4% PFA prepared in ice-cold PBS for 20 min at room temperature. After washing thrice with 0.3% PBST for 30 min, secondary antibodies were applied and washed, and tissues were mounted according to standard protocols (Sharma et al., 2019).

### Antibody production

Total RNA was isolated from third instar larvae and cDNA was synthesised. A portion of the *Ppn* gene, corresponding to amino acids 112-266, was amplified from the cDNA pool by PCR using the following primers: 5′-AAAACCATGGAGGAAGAATCCGACTTCCG-3′ (forward) and 5′-AAATGCGGCCGCGTTCAGGTAGTAGTGTCCAC-3′ (reverse) for N-terminal His tagging. The resulting fragments were subcloned into the vector pET29a for expression in *E. coli* (BL21). His-tagged proteins were purified using a Ni-NTA column. Polyclonal antibodies against the purified Ppn peptide were raised in guinea pigs by Young In Frontier (Seoul, Korea). The immunization process involved two guinea pigs, and the production of antibodies followed standard protocols. Antiserum generated from the N-terminal His-tagged fusion construct was used primarily.

Antibody validation was performed using two methods. First, co-staining with the previously established rabbit anti-Ppn antibody was conducted to confirm the specificity of the guinea pig anti-Ppn antibody (Fig. S3). Second, western blotting was performed on Ppn knockdown larvae to validate the antibody's effectiveness in detecting Ppn protein expression (Fig. 4O).

### Quantification of lymph gland phenotypes

To measure lobe volume, the sum of the areas of all slices was multiplied by 4 μm, the interval between *z*-stack slices. Lobe areas were quantified using ImageJ software. Confocal *z*-stack images were converted to 8-bit and a threshold was set. Regions of interest (ROIs) were defined using the freehand selections tool. Secondary lobe areas were measured using fluorescence microscopy (Zeiss Axio Imager 2) with ROIs defined in ImageJ.

To calculate the ratio of P1-positive and dome^MESO^-GFP-positive cells, three *z*-stack images (top, middle and bottom) were analysed. Total cell numbers in each section were determined by DAPI staining. The number of P1-positive or dome^MESO^-GFP-positive cells within the defined ROIs was counted. The ratio of positive cells for each type was calculated by dividing the number of positive cells by the total cell count for each ROI, and the average percentage across all sections was computed to obtain the final index. For the ProPOA1.sGFP-positive cell index, the percentage of the positive area relative to the total area was calculated for each *z*-section image and averaged. Other differentiation indices (P1, *GAL4*-driven GFP-positive cells, dome^MESO^-GFP and DE-cad) were quantified using ImageJ. Confocal images were converted to 8-bit, a threshold was set, and ROIs were defined using the freehand selections tool. Area fraction was then calculated. Detailed sample sizes are shown in dotted plots. Mys-positive cells were counted manually in primary lobes of mid-, late- or wandering third instar larvae using a fluorescence microscope. Both small and enlarged Mys-positive cells were significantly increased in *Hml*Δ*>Ppn-i* larvae compared to controls, with the latter shown in Fig. 2I. The total number of pH3-positive cells per primary lobe or posterior lobes of mid-third instar larvae was counted, as well as dpERK-positive cells per primary or posterior lobes of wandering third instar larvae. All staining experiments were performed independently at least three times, with at least ten lobes analysed per genotype per experiment.

To measure the ratio of lymph gland rupture, lymph glands were classified using bright-field microscopy into three groups (Louradour et al., 2017): ‘intact’ (lobe margin unimpaired), ‘dispersing’ (lobe border discontinuous, with some cells escaping) and ‘dispersed’ (lobe absent or barely remained). The percentage of the lymph gland dispersal was calculated by dividing the number of glands in each category by the total number of lymph glands. At least 20 lobes per genotype were analysed.

### Quantification of the ECMs

To measure relative ECM intensity in lymph glands, 200× confocal images were analysed for fluorescence intensity. The lymph gland was traced with a freehand selection brush in ImageJ. The fluorescence intensity of Ppn (IntDen) in the ROI was divided by the mean intensity of control samples. Each experiment was performed independently at least three times, analysing at least ten lobes per genotype.

For Ppn intensity at intercellular contacts in fat bodies, 400× confocal images were analysed as described (Dai et al., 2017). The cellular edges marked by phalloidin were traced with 4 μm freehand selection brush in ImageJ and the same procedures were followed.

ECM branches in the MZ were quantified as previously described (Khadilkar et al., 2020). Confocal images were converted to 8-bit, and a threshold was applied. The ‘Vessel Analysis’ plugin (Vascular Density) of ImageJ was used to define the dome^MESO^-GFP-positive area with the freehand selections tool. Each experiment was performed independently at least three times, analysing at least 11 lobes per genotype.

### Intensity plot graphs of the ECMs

To demonstrate the colocalisation of Ppn and other ECM components within the lymph gland, 200× confocal images were analysed. A line was drawn across the lymph gland using the ‘Straight line’ tool in ImageJ to define the ROI, ensuring it traversed ECM branches in the MZ and cellular edges in the CZ. Three arrowheads and one asterisk were marked on the corresponding confocal images and the intensity plot graphs (Fig. S5B,C,E,F,H,I,K,L). The left arrowhead represents the outer sheath of the lymph gland adjacent to the MZ, the middle arrowhead indicates the boundary between the MZ and CZ, and the right arrowhead marks the outer sheath adjacent to the CZ. The asterisk highlights the most pronounced difference in fluorescence intensity between ECM and Ppn within the CZ. Fluorescence intensity profiles were generated using the ‘RGB Profiler’ plugin of ImageJ, with intensity lines for each ECM component colour-coded to match the corresponding confocal image.

### Bacterial infection and wasp infestation

*E*. *coli* was grown in Luria–Bertani broth containing ampicillin (100 mg/ml) overnight at 37°C. Cultures were centrifuged at 6000 ***g*** for 15 min, and the pellet was washed with 10% sucrose. *Drosophila* larvae at 84 h AEL (for 36 hpi experiments) or 96 h AEL (for 24 hpi experiments) were washed with ddH_2_O and 70% ethanol, then pricked at the posterolateral body wall with a tungsten needle (10130-05, Fine Science Tools) previously coated with the bacterial solution. After infection, larvae were transferred to cornmeal-agar medium on humified filter paper at 25°C until dissection. Control larvae underwent the same procedure without injury/infection. For a clean injury control, a 10% sucrose solution replaced the bacterial pellet. For wasp infestation, *Drosophila* larvae at 96 h AEL were exposed to *Leptopilina boulardi* G486 for 6 h at 25°C. Larvae were then allowed to develop at 25°C and dissected 24 or 48 h post-parasitism. Uninfected controls were processed similarly.

### Statistical analyses

Experiments were conducted at least three times. Melanotic masses counts were graphed using Microsoft Excel with standard errors of the mean. Other graphs, including scatter plots and diagrams, were created using GraphPad Prism 5. Statistical analyses for quantification of lymph gland rupture were performed using the Pearson's Chi-squared test in Excel. For other quantifications, mean±s.d. is shown and significance tests by *t*-test (Mann–Whitney non-parametric test) were performed using Prism 5. The thresholds for statistical significance were set at *P<0.05, **P<0.01 and ***P<0.001.

## Supplementary Material

10.1242/develop.204367_sup1 Supplementary information

Table S1. An RNAi-based screen for genes involved in melanotic mass formation. *Cg-GAL4 UAS-mys-RNAi* flies were used as a sensitised genetic background. Genes marked in red were identified as enhancers of melanotic mass formation, while those marked in blue were identified as suppressors. BDSC, The Bloomington *Drosophila* Stock Center, USA; NIG, National Institute of Genetics, Japan; VDRC, Vienna *Drosophila* RNAi Center, Austria.
