# Supplementary material for: The matrix glycoprotein Papilin maintains the haematopoietic progenitor pool in Drosophila lymph glands
Source: Development. 2025 Apr 10;152(7):dev204367. doi: 10.1242/dev.204367 (PMC12045604; doi:10.1242/dev.204367)
Supplement: Supplementary information [file develop-152-204367-s1.pdf]

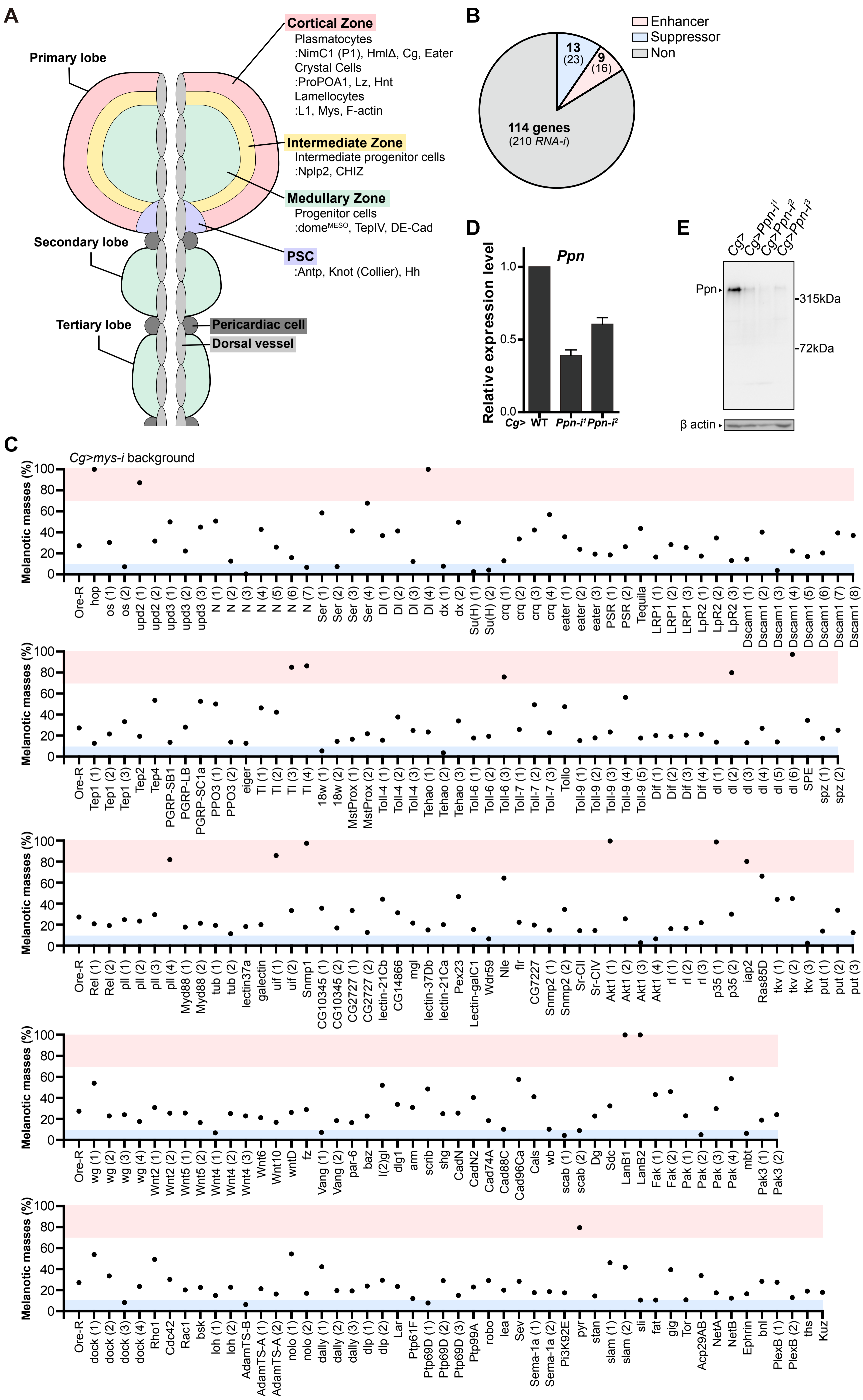

Development • Supplementary information

Fig. S1. Genetic screen identifying modifiers of melanotic mass formation. (A) Schematic diagram of the lymph gland. (B) Diagram showing the numbers of genes and *RNA-i* lines (latter in parentheses) isolated as enhancers/suppressors. (C) Results of the RNA interference (RNAi)-based genetic screen, isolating enhancers (red) and suppressors (blue) of the melanotic mass phenotype of *Cg-GAL4 UAS-mys-RNAi* (*Cg>mys-i*), a sensitised genetic background. (D,E) Knockdown efficiency was analysed via qRT-PCR (D) and western blotting (E), respectively, using whole larval lysates. For western blotting, rabbit anti-Ppn antisera were used. β-actin was used as a loading control.

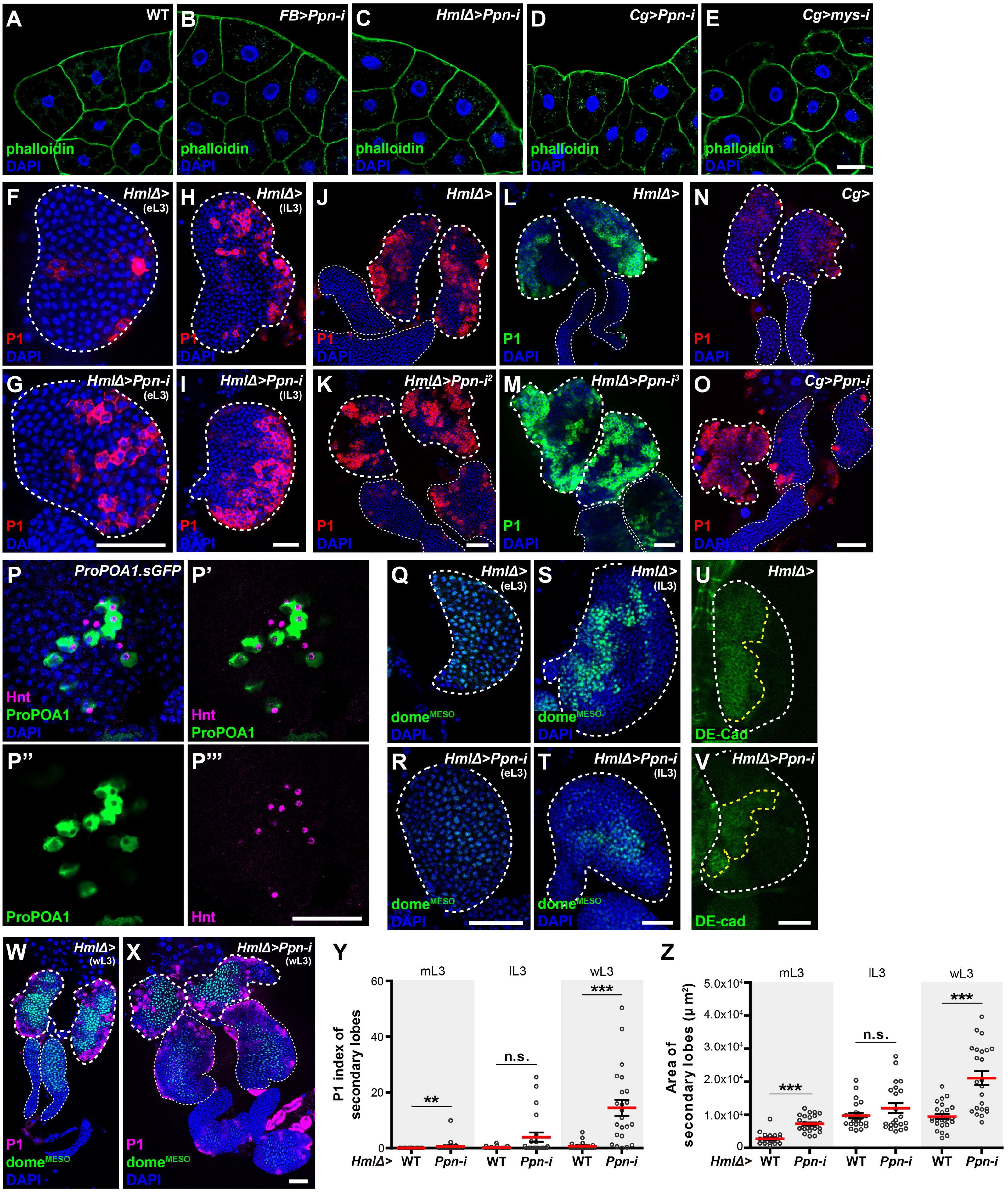

Fig. S2. Haemocyte differentiation was analysed in the lymph glands of *Ppn*-knockdown larvae (related to Fig. 2).

(A-E) Confocal images of fat bodies of the indicated genotypes. F-actin was stained with FITC-phalloidin (green) and cell nuclei were stained with DAPI (blue). (F-O) Immunohistochemistry was performed to visualise plasmatocytes using the P1 antibody in red (F-K,N,O) or green (L,M). Cell nuclei were stained with DAPI in blue. *HmlΔ-GAL4/+* (F,H,J,L) or *Cg-GAL4/+* (N) were used as controls. (G,I) *HmlΔ>Ppn-i*^{1}. (K) *HmlΔ>Ppn-i*^{2}. (M) *HmlΔ>Ppn-i*^{3}. (O) *Cg>Ppn-i*^{1}. Primary and

Development • Supplementary information

secondary lobes were demarcated with thick and thin dashed lines, respectively. (P-P''') To verify whether ProPOA1.sGFP marks the crystal cells, lymph glands of *ProPOA1.sGFP* larvae were stained with the anti-Hnt antibody in magenta. ProPOA1.sGFP was expressed in nearly all Hnt-positive cell. (Q-V) Prohaemocytes were labeled with dome^{MESO}-GFP (Q-T) and the medullary zone (MZ) was stained with the anti-DE-cad antibody (U,V; green) in *HmlΔ-GAL4/+* controls (Q,S,U) and *HmlΔ>Ppn-i* (R,T,V). Cell nuclei were stained with DAPI in blue (Q-T). The Primary lobe and MZ were demarcated with white- and yellow-dashed lines, respectively in (U,V). (W,X) Confocal tile-scan images of lymph glands. Plasmatocytes were stained with the P1 antibody (magenta), prohaemocytes were marked with dome^{MESO}-GFP (green), and cell nuclei were stained with DAPI (blue). Scale bars: 50 µm. (Y,Z) Quantification of P1-positive cell index per secondary lobe (Y) and the size of the secondary lobe (Z) are presented. n.s., not significant; Mann-Whitney *U* test was used.

Development • Supplementary information

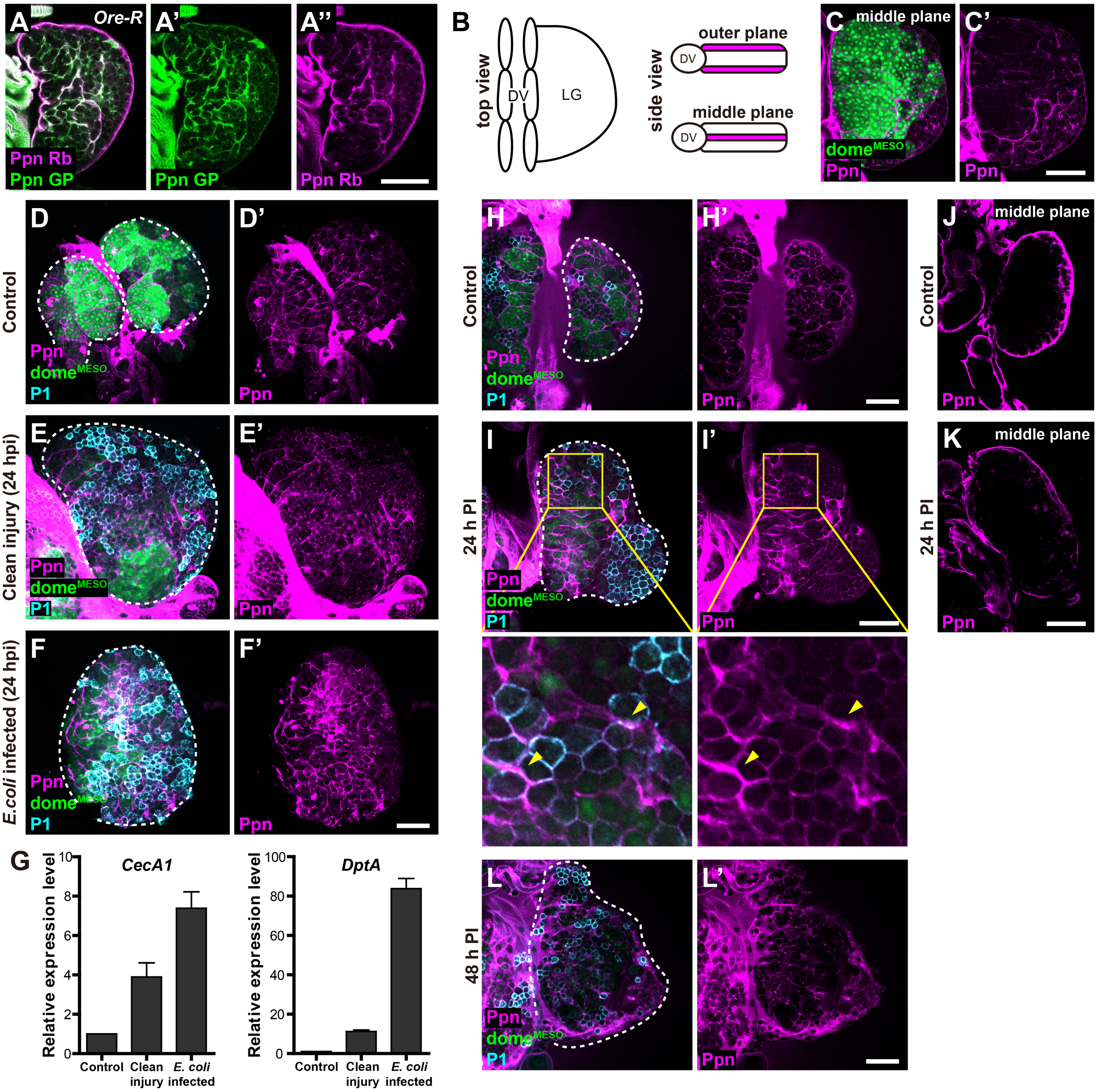

Fig. S3. Distribution of Ppn in larval lymph glands under normal and infection conditions (related to Fig. 3). (A-A'') Specificity of the two different anti-Ppn antisera was assessed via co-staining. Guinea pig antisera (Ppn GP in green) and rabbit antisera (Ppn Rb in magenta) were used. (B) Cartoons depicting the top view (*left*) or side views (*right*) of the dorsal vessel (DV) and lymph gland (LG). Outer plane refers to the confocal sections taken at the top or bottom, while middle plane refers to the confocal sections in the inner region of the lymph glands. (C,C') Distribution of Ppn in the middle plane of late-third instar larval lymph glands was visualised using anti-Ppn antisera (magenta). Prohaemocytes were marked with dome^{MESO}-GFP (green). (D-F') Confocal images of the lymph glands of *dome^{MESO}-GFP* larvae at 120 h AEL. (D,D') Larvae without injury. (E,E') Larvae with clean injury treated with 10 % sucrose at 24 h post-injury (hpi). (F,F') Larvae infected with *E. coli* at 24 hpi. Ppn and prohaemocytes were marked as described in (C,C'), and plasmatocytes were stained with the P1 antibody (cyan). (G) Induction of the antimicrobial peptide genes *Cecropin A1* (*CecA1*) and *Diptericin* (*Dpt*) was examined via qRT-PCR under the indicated conditions. Larvae from control groups, those with clean injuries at 24 hpi, and those infected with *E. coli* at 24 hpi were collected at

Development • Supplementary information

120 h AEL for expression analysis. (H-L') Confocal images of the lymph glands of *dome*^{MESO}*-GFP* larvae. (H,H',J) Controls. (I,I',K) 24 h, and (L,L') 48 h post wasp infestation (PI). Ppn, plasmatocytes, and prohaemocytes were stained as described in (D-F'). Higher magnification views of the regions outlined by yellow squares in (I,I') are shown below. Primary lobes were demarcated with white dashed lines. (J,K) Distribution of Ppn in the middle plane of the lymph glands was visualised using anti-Ppn antisera (magenta). Scale bars: 50 µm.

Development • Supplementary information

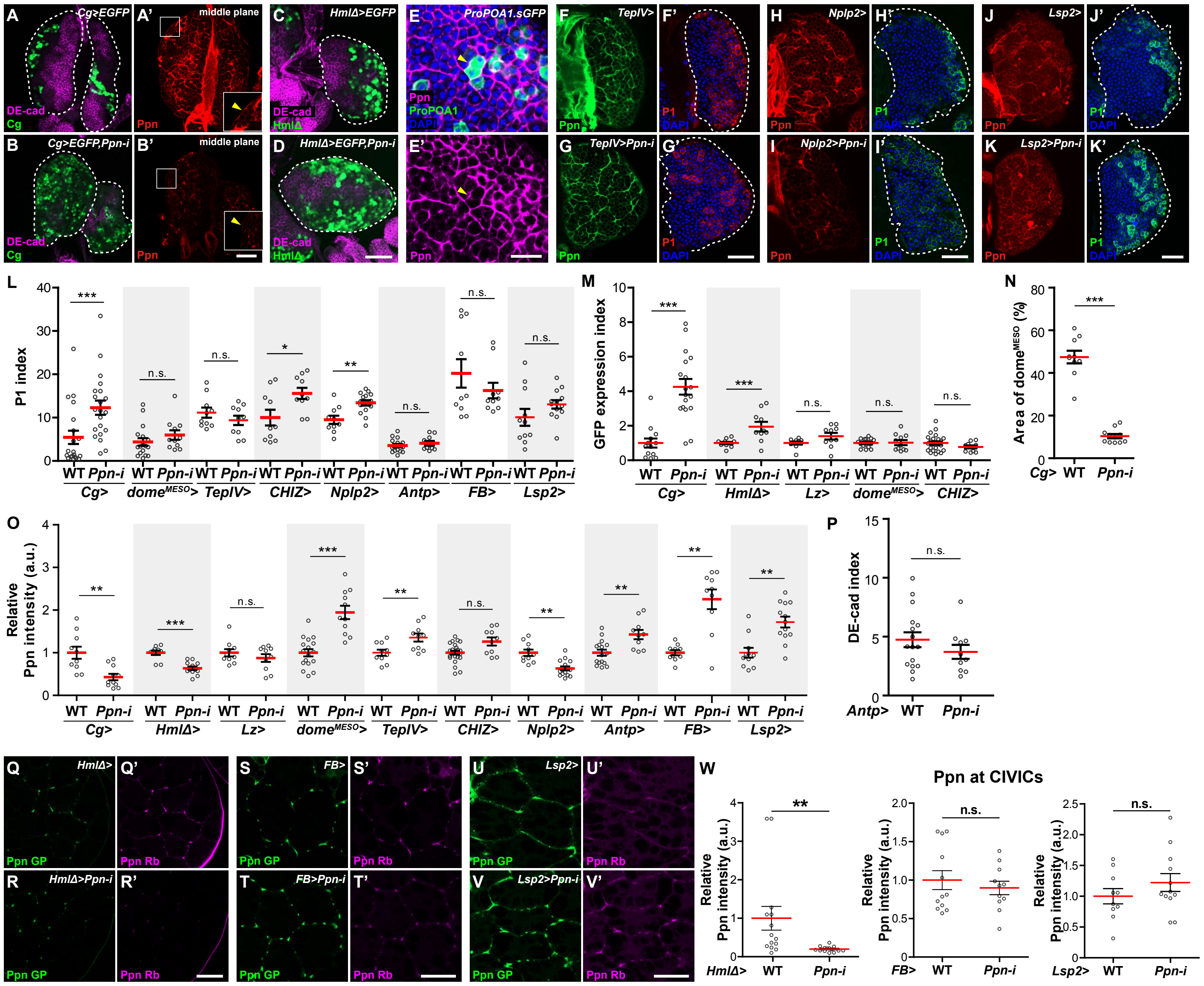

Fig. S4. Analysis of Ppn distribution in lymph glands and fat bodies upon knockdown with tissue-specific drivers (related to Fig. 4). (A-D) Changes in the distribution of haemocyte differentiation and outermost Ppn were analysed in the lymph glands of late-third instar larvae of the indicated genotypes. *Cg>EGFP* (A-B') or *HmlΔ>EGFP* (C,D) were crossed with *Ore-R* for controls or with *UAS-Ppn-i*. MZ was stained with DE-cad in magenta, and *Cg-GAL4*-driven EGFP-positive cells (A,B) or *HmlΔ-GAL4*-driven EGFP-positive cells (C,D) were marked in green, respectively. The distribution of Ppn in the middle plane of the lymph glands was visualised using anti-Ppn antisera (red; A',B'). The enlarged section corresponding to the region enclosed within the white box shows the outermost Ppn membrane, marked with a yellow arrowhead. (E,E') Ppn distribution in lymph glands of *ProPOA1.sGFP* larvae at the wandering-third instar stage was visualised using anti-Ppn antisera in magenta. Crystal cells were marked with ProPOA1.sGFP in green, and cell nuclei were stained with DAPI in blue. Yellow arrowhead indicates the absence of Ppn around ProPOA1.sGFP-positive cells. (F-K') Changes in protein levels and distribution of Ppn and haemocyte differentiation were analysed in the lymph glands of wandering-third instar larvae of the indicated genotypes. *TepIV-GAL4* (F-G'), *Nplp2-GAL4* (H-I'), or *Lsp2-GAL4* (J-K') were crossed with *Ore-R* for controls or *UAS-Ppn-i* for

Development • Supplementary information

tissue-specific knockdown. Ppn was stained with anti-Ppn antisera in green (F,G) or red (H,I,J,K), plasmatocytes were stained with the P1 antibody in red (F',G'), or green (H',I',J',K'), and cell nuclei were stained with DAPI in blue. Primary lobes were demarcated with white dashed lines. (L-P) Quantification of P1-positive cell index (L), GFP expression cell index (M), dome^{MESO}-GFP-positive cell index (N), relative Ppn fluorescence intensity in the lymph glands (O), and DE-cad-positive cell index (P). (Q-V') Changes in Ppn levels in the larval fat bodies of the indicated genotypes were analysed via immunohistochemistry. *HmlΔ-GAL4* (Q-R'), *FB-GAL4* (S-T'), or *Lsp2-GAL4* (U-V') were crossed with *Ore-R* for controls or *UAS-Ppn-i* for tissue-specific knockdown. Ppn were stained with Ppn GP in green or Ppn Rb in magenta. Scale bars: 50 µm in A-D', F-K', and Q-V'; 20 µm in E,E'. (W) Quantification of the relative intercellular Ppn fluorescence intensity in fat bodies of the indicated genotypes. n.s., not significant; Mann-Whitney *U* test was used.

Development • Supplementary information

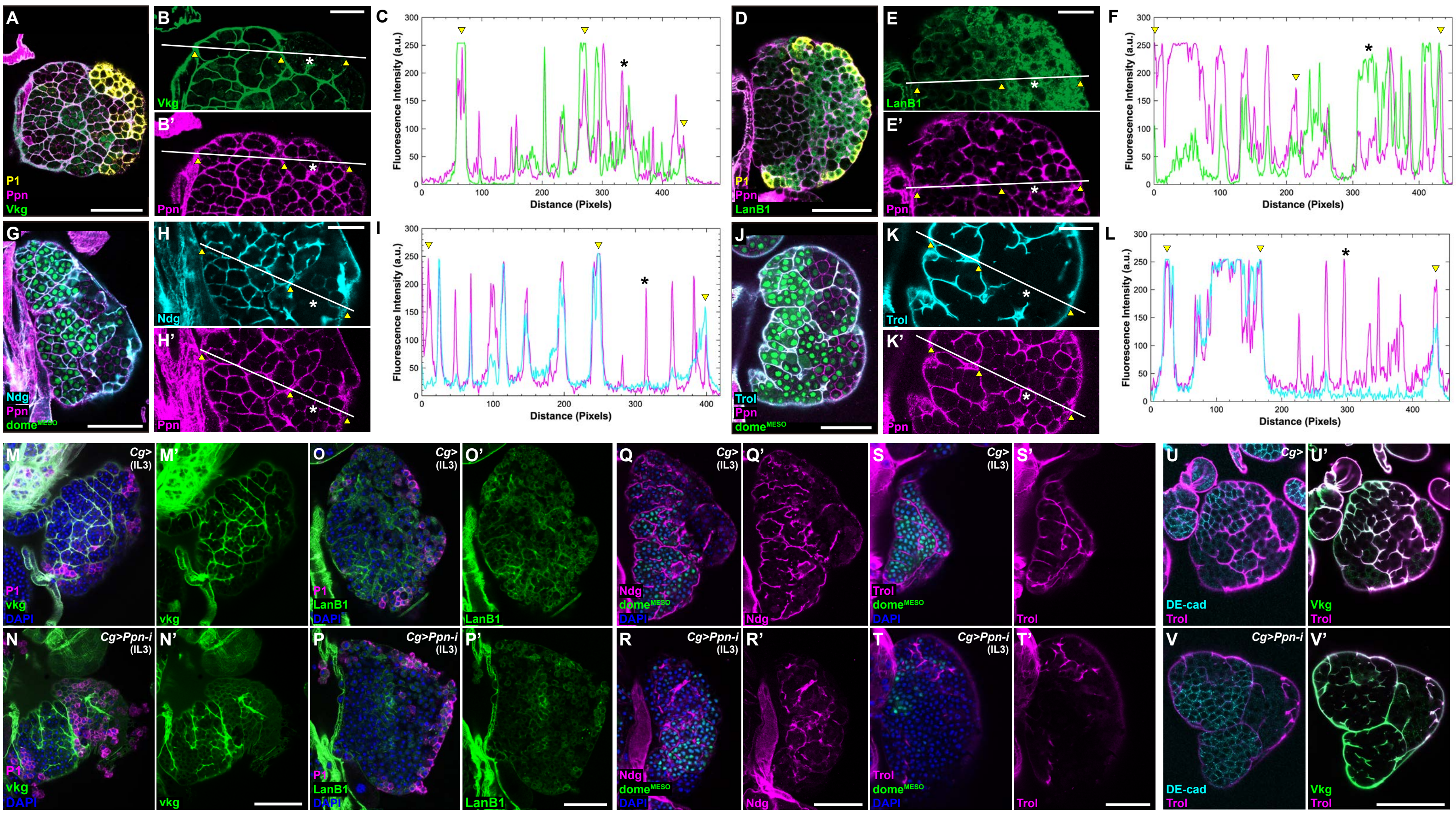

Fig. S5. Analysis of the breakdown of the major ECM components in *Ppn*-knockdown larvae (related to Fig. 5). (A-L) Confocal images and fluorescence intensity plot graphs illustrating ECM lamellae and Ppn distribution in the lymph glands of mid-third instar larval. The distribution of collagen (visualised by Vkg-GFP in green; A-B'), laminin (LanB1.sGFP in green; D-E'), nidogen (anti-Ndg antisera in cyan; G-H'), and Trol (anti-Trol antisera in cyan; J-K') was analysed and compared to that of Ppn (anti-Ppn antisera in magenta). Plasmatocytes were stained with P1 antibody in yellow (A,D) and prohaemocytes were visualised using dome^{MESO}-GFP in green (G,J). Fluorescence intensity plot graphs comparing ECM and Ppn along the MZ-CZ axis are shown in (C), (F), (I), and (L), corresponding to (B,B'), (E,E'), (H,H'), and (K,K'), respectively. The left, middle, and right arrowheads mark the innermost MZ, the MZ-CZ boundary, and the outermost CZ area, respectively. The asterisk indicates the strongest fluorescence intensity difference between ECM and Ppn in the CZ. (M-T') Confocal images of the major ECM components in the lymph glands of *Cg-GAL4* control or *Cg>Ppn-i* larvae during the late-third instar stage larvae were analysed. Collagen (green; M-N'), laminin (green; O-P'), nidogen (magenta; Q-R'), and Trol (magenta; S-T') were visualised as described above. Plasmatocytes were stained with P1 antibody in magenta (M,N,O,P), and MZ was visualised using dome^{MESO}-GFP in green (Q,R,S,T). Cell nuclei were stained with DAPI in blue. (U-V') Changes in Trol distribution were analysed by immunohistochemistry in *Cg-GAL4* controls (U,U') and *Cg>Ppn-i* larvae (V,V'). Trol was stained with anti-Trol antisera in magenta. MZ was stained with anti-DE-cad antibody in cyan (pseudo-colour), and collagen was visualised by Vkg-GFP in green. Scale bars: 50 μm.

Development • Supplementary information

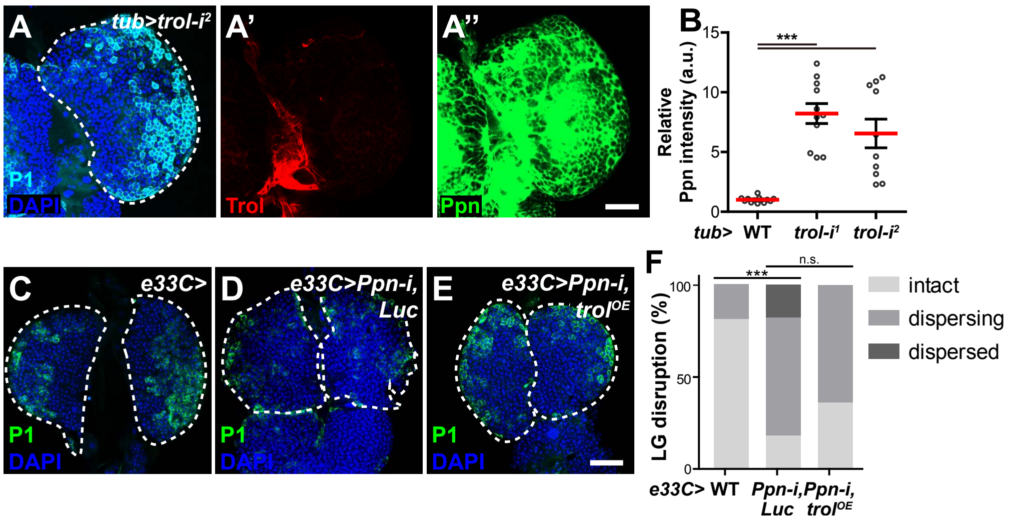

Fig. S6. Overexpression of Trol partially rescues *Ppn*-knockdown induced defects in the lymph gland (related to Fig. 6). (A-A") Distribution of Trol (anti-Trol antisera; red) and Ppn (anti-Ppn antisera; green) following *trol* knockdown. Plasmatocytes were stained with the P1 antibody (cyan), and cell nuclei were stained with DAPI (blue). (B) Quantification of relative Ppn fluorescence intensity in the lymph glands of the indicated genotypes. Mann-Whitney *U* test was used. (C-E) Suppressive effects of *trol* overexpression on *Ppn*-knockdown phenotypes were analysed. *e33C-GAL4* was crossed with the following three genotypes: *Ore-R*; *UAS-Ppn-i,Luc*; and *UAS-Ppn-i,trol^{OE}*. Plasmatocytes were stained with the P1 antibody (green), and cell nuclei were stained with DAPI (blue). Scale bars: 50 µm. (F) Quantification of the dispersal of late-third instar larval lymph gland, presented as percentile. At least 22 lobes were measured for each genotype. n.s., not significant; Pearson's Chi-squared test was used.

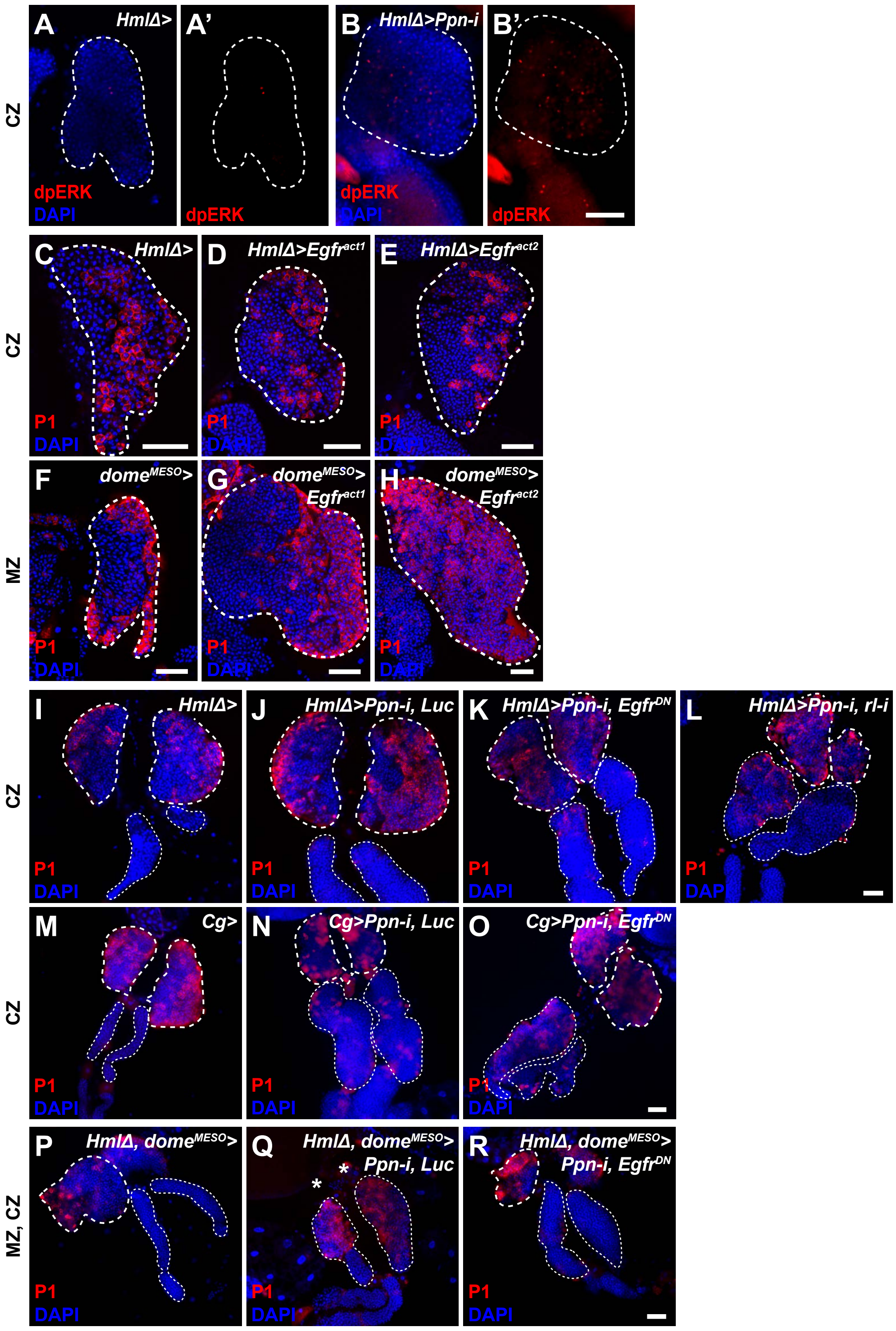

Fig. S7. Analysis of the roles of the EGFR pathway in larval lymph glands (related to Fig. 7). (A-B') Activity of the EGFR pathway was monitored in the lymph glands of *HmlΔ-GAL4*/+ control (A,A') and *HmlΔ>Ppn-i* (B,B') larvae via immunostaining using the anti-dpERK antibody in red. Cell nuclei were stained with DAPI in blue. (C-H) Effects of EGFR pathway activation were analysed in lymph glands. The EGFR pathway was selectively activated in CZ using *HmlΔ-GAL4* (C-E) or MZ using *dome*^{MESO}*>GFP* (F-H). Plasmatocytes were stained with the P1 antibody (red), and cell nuclei were stained with DAPI (blue). (I-O) EGFR pathway activation was suppressed in CZ using *HmlΔ-GAL4* (I-L) or *Cg-GAL4* (M-O) in *Ppn*-knockdown larvae. *HmlΔ-GAL4* or *Cg-GAL4* were crossed with *Ore-R* (I,M), *UAS-Ppn-i, Luc* (J,N), *UAS-Ppn-i, Egfr*^{DN} (K,O), or *UAS-Ppn-i, rl-i* (L). Plasmatocytes and cell nuclei were stained as described in (C-H). (P-R) Suppressive effects of the EGFR pathway blockade on *Ppn*-knockdown larvae were confirmed using a combined *GAL4* line active in both CZ and MZ. *HmlΔ-GAL4; dome*^{MESO}*>GFP* was crossed with *Ore-R* (P), *UAS-Ppn-i, Luc* (Q), or *UAS-Ppn-i, Egfr*^{DN} (R). Plasmatocytes and cell nuclei were stained as described in (C-H). Primary and secondary lobes were demarcated with thick and thin dashed lines, respectively. Asterisks in (Q) indicate the position of the completely dispersed primary lobes. Scale bars: 50 µm.

Development • Supplementary information

Table S1. An RNAi-based screen for genes involved in melanotic mass formation. *Cg-GAL4 UAS-mys-RNAi* flies were used as a sensitised genetic background. Genes marked in red were identified as enhancers of melanotic mass formation, while those marked in blue were identified as suppressors. BDSC, The Bloomington *Drosophila* Stock Center, USA; NIG, National Institute of Genetics, Japan; VDRC, Vienna *Drosophila* RNAi Center, Austria.

Available for download at
https://journals.biologists.com/dev/article-lookup/doi/10.1242/dev.204367#supplementary-data

Development • Supplementary information
